# Structural and immunochemical relatedness suggests a conserved pathogenicity motif for secondary cell wall polysaccharides in *Bacillus anthracis* and infection-associated *Bacillus cereus*

**DOI:** 10.1371/journal.pone.0183115

**Published:** 2017-08-23

**Authors:** Nazia Kamal, Jhuma Ganguly, Elke Saile, Silke R. Klee, Alex Hoffmaster, Russell W. Carlson, Lennart S. Forsberg, Elmar L. Kannenberg, Conrad P. Quinn

**Affiliations:** 1 Centers for Disease Control and Prevention, Atlanta, GA, United States of America; 2 Complex Carbohydrate Research Center, University of Georgia, Athens, GA, United States of America; 3 Indian Institute of Engineering Science and Technology, Shibpur, West Bengal, India; 4 Robert Koch-Institute, Center for Biological Threats and Special Pathogens, Berlin, Germany; Laboratory of Bacterial Pathogenesis and Immunology, UNITED STATES

## Abstract

*Bacillus anthracis* (*Ba*) and human infection-associated *Bacillus cereus* (*Bc*) strains *Bc* G9241 and *Bc* 03BB87 have secondary cell wall polysaccharides (SCWPs) comprising an aminoglycosyl trisaccharide repeat: →4)-β-d-Man*p*NAc-(1→4)-β-d-Glc*p*NAc-(1→6)-α-d-Glc*p*NAc-(1→, substituted at GlcNAc residues with both α- and β-Gal*p*. In *Bc* G9241 and *Bc* 03BB87, an additional α-Gal*p* is attached to O-3 of ManNAc. Using NMR spectroscopy, mass spectrometry and immunochemical methods, we compared these structures to SCWPs from *Bc* biovar *anthracis* strains isolated from great apes displaying “anthrax-like” symptoms in Cameroon (*Bc* CA) and Côte d’Ivoire (*Bc* CI). The SCWPs of *Bc* CA/CI contained the identical HexNAc trisaccharide backbone and Gal modifications found in *Ba*, together with the α-Gal-(1→3) substitution observed previously at ManNAc residues only in *Bc* G9241/03BB87. Interestingly, the great ape derived strains displayed a unique α-Gal-(1→3)-α-Gal-(1→3) disaccharide substitution at some ManNAc residues, a modification not found in any previously examined *Ba* or *Bc* strain. Immuno-analysis with specific polyclonal anti-*Ba* SCWP antiserum demonstrated a reactivity hierarchy: high reactivity with SCWPs from *Ba* 7702 and *Ba* Sterne 34F2, and *Bc* G9241 and *Bc* 03BB87; intermediate reactivity with SCWPs from *Bc* CI/CA; and low reactivity with the SCWPs from structurally distinct *Ba* CDC684 (a unique strain producing an SCWP lacking all Gal substitutions) and non-infection-associated *Bc* ATCC10987 and *Bc* 14579 SCWPs. *Ba*-specific monoclonal antibody EAII-6G6-2-3 demonstrated a 10–20 fold reduced reactivity to *Bc* G9241 and *Bc* 03BB87 SCWPs compared to *Ba* 7702/34F2, and low/undetectable reactivity to SCWPs from *Bc* CI, *Bc* CA, *Ba* CDC684, and non-infection-associated *Bc* strains. Our data indicate that the HexNAc motif is conserved among infection-associated *Ba* and *Bc* isolates (regardless of human or great ape origin), and that the number, positions and structures of Gal substitutions confer unique antigenic properties. The conservation of this structural motif could open a new diagnostic route in detection of pathogenic *Bc* strains.

## Introduction

*Bacillus anthracis* is a Gram-positive, spore forming bacterium that causes anthrax. It is considered a high threat bioterrorism agent because of the relative ease with which its highly resilient spores can be weaponized and dispersed [1–4]. Renewed interest in developing rapid diagnostic tools and effective medical countermeasures against anthrax was generated subsequent to the anthrax letter attacks in 2001 [5,6]. We sought to determine whether carbohydrates located on the *B*. *anthracis* (Ba*)* vegetative cell have structural or immunochemical properties suitable for the development of new and improved medical counter-measures, such as improved diagnostics, vaccines and targeted immunotherapies.

*B*. *anthracis* is a member of the *B*. *cereus* (*Bc*) group of related species that also includes *B*. *thuringiensis*. These species show high DNA sequence similarities, but display an array of pathogen and non-pathogen associated phenotypes [7–9]. The considerable variability in their infection-associated properties and host specificity has primarily been attributed to differences in the plasmid content [10].

**Fig 1:**
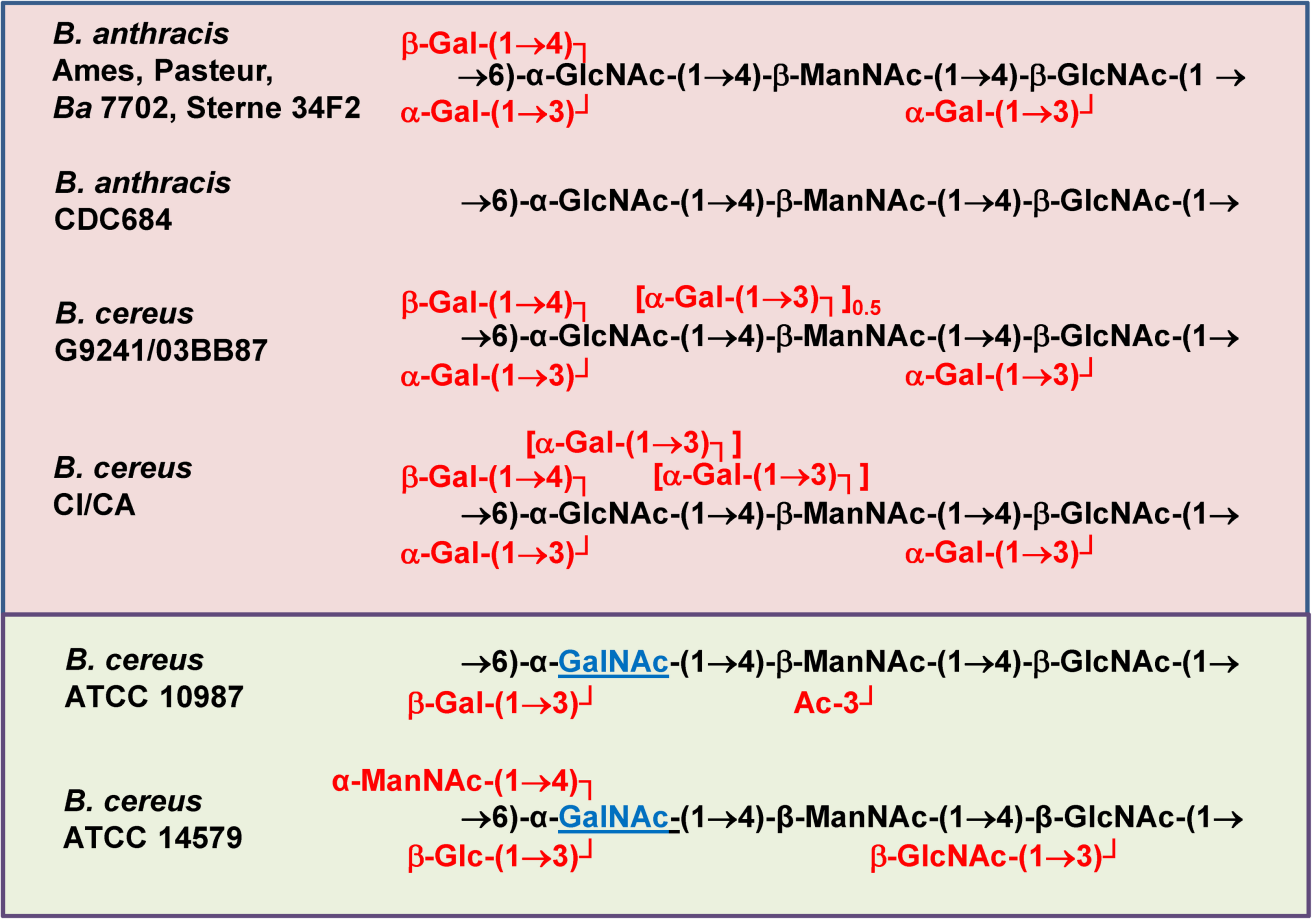
Currently known secondary cell wall polysaccharide (HF-SCWP) structures of strains from the *B*. *cereus* group. HF-released SCWPs from infection-associated *B*. *anthracis* and *B*. *cereus* are shown with red background. The HF-SCWP from the environmental *B*. *cereus* strains have a green background. The conserved aminogycosyl backbone residues are shown in black; the dissenting GalNAc residues found in the repeating unit of the environmental *B*. *cereus* isolates are shown in blue. The *Ba* CDC684 strain lacks all Gal substituents. For further *Bacillus* strain characterization, see **Table 1**.

We have previously reported that the structure and composition of HF-released secondary cell wall polysaccharides (HF-SCWPs) from *Ba* vegetative cells are not dependent on plasmid content [11], are specific for *Ba* and antigenic [12–14]. For example, antisera raised against live and killed *Ba* spores react with the HF-SCWP from *Ba*, but not with the HF-SCWPs from *Bc* ATCC 10987, a dairy isolate, or from the type strain *Bc* ATCC 14579 [11–14]. In an immunological survey with a polyclonal antiserum raised against *Ba* HF-SCWPs [14], we demonstrated qualitative serological cross-reactivity with cells and isolated cell walls from *Bc* strains capable of causing severe or fatal illness in humans (e.g. *Bc* G9241 and 03BB87 causing severe pneumonia) but not with closely related *Bc* cells and cell walls from non-infection-associated strains, such as *Bc* ATCC 10987 and *Bc* ATCC 14579. This observation suggested a degree of antigenic structure conservation between SCWPs from infection-associated *Ba* and *Bc* strains. The repeating unit structures for HF-SCWPs from all currently examined Bc group members are summarized in **Fig 1**.

We investigated the SCWP structures of two recently isolated *Bc* strains, strain *Bc* CI from Côte d’Ivoire and strain *Bc* CA from Cameroon designated as *B*. *cereus* biovar *anthracis*, that caused “anthrax-like” disease in great apes [15–17]. These specific *B*. *cereus* strains have recently been identified as the causative agents of severe outbreaks of anthrax-like disease among a diversity of great apes in the African tropical rain forest environment (see Hoffmann C, Zimmermann F, *et al*., *Nature* 548, 82–99 (2017). We compared these structures to those from infection-associated and non-infection-associated *Bc* group members. Our findings show that the HF-SCWPs of both great ape strains contained the identical HexNAc trisaccharide backbone core structure and Gal modifications known from *Ba* HF-SCWP. In addition, their SCWP contains the additional Gal-substitution at 50% of ManNAc residues originally identified in human infection-associated *Bc* strains G9241/03BB87/03BB102. Interestingly, both great ape-derived strains contained an additional, unique disaccharide substitution at some of the ManNAc residues that varied in percentage between them. The data from strains *Bc* CI and CA support the hypothesis of a conserved structural core motif of SCWPs in virulent, infection-associated *Bc* group strains. We also describe a comparison of the immunoreactivity of these SCWPs with both *Ba* SCWP polyclonal antiserum (pAb) and monoclonal antibody (mAb) EAII-6G6-2-3 specific for *Ba* [18,19]. The results corroborate the structural data and further support the existence of an antigenically distinct and structurally conserved motif in the SCWPs of disease-causing members of the *Bc* group of related species.

## Methods

### Bacterial strains and clinical isolates

The strains and isolates used in this study are listed in **Table 1**.

**Table 1:** *Bacillus anthracis* and *B*. *cereus* strains used in this study.

Strain	Source	Clinical information	Reference
*B*. *anthracis* Sterne 34F2	Bovine isolate, South Africa, CDC collection	Veterinary vaccine	[20]
*B*. *anthracis* 7702	Institute Pasteur	Sterne strain derivative	[21]
*B*. *anthracis* CDC684	CDC collection	Attenuated strain that was misclassified as *B*. *megaterium*	[19]
*B*. *cereus* ATCC 10987	Dairy isolate (1930, Canada)	N/A^a^	[22]
*B*. *cereus* ATCC 14579	Type strain, CDC collection	N/A	[23]
*B*. *cereus* G9241	Human blood isolate (1994, Louisiana)	Severe pneumonia	[24]
*B*. *cereus* 03BB87	Human blood isolate, (2003, Texas)	Fatal pneumonia	[22]
*B*. *cereus* CI (*Bc* biovar *anthracis* CI)	Great ape isolate, Côte d’Ivoire	Fatal infection in great apes	[16,17]
*B*. *cereus* CA (*Bc* biovar *anthracis* CA)	Great ape isolate, Cameroon	Fatal infection in great apes	[16,17]

^a^N/A = not available

### Use and care of animals

All animal procedures were carried out in strict accordance with recommendations in the Guide for the Care and Use of Laboratory Animals of the National Institutes of Health. Specifically, procedures for antisera production in rabbits were approved by the Institutional Animal Care and Use Committee (IACUC) of the Centers for Disease Control and Prevention (protocol #1420SAIRABC-A1). The rabbits were housed in stainless steel cages. Room temperature was maintained in a range from 20 to 23°C; the light/dark cycle was approximately 12 hours each per d. Water was available to the animals *ad libitum* and environmental enrichment was provided e. g. with treats and toys. Rabbits were ultimately euthanized by exsanguination under anesthesia. There was no direct use of non-human primates *per se* in our current study; all *Bacillus* strains used in this study (**Table 1**) originally isolated from the great apes were previously described by Klee *et al*., [15,16].

### Isolation of cell walls from *Bc* CA and *Bc* CI vegetative cells

Cell walls were prepared from cultured cells as previously described [12,14] following the modified procedure as described [25]. The procedure yielded approximately 15–35 mg of cell walls per liter of cultured cells.

### Isolation and purification of SCWP from *Bc* CA and *Bc* CI

The SCWPs examined in this study, and their parent strains, are summarized in **Table 1**and **Fig 1**(adapted from [12,13,26,27] and unpublished results). The SCWPs, bound to peptidoglycan via phosphate, were released from the cell walls by treatment with 48% aqueous hydrofluoric acid (HF) and purified as previously described [12,14,26,27]. Briefly: the cell wall preparations from the *Bc* isolates from great apes (strains *Bc* CA and *Bc* CI) were treated with 48% HF at 4°C for 48 h. The reaction mixtures were rotated slowly in an end-over end mixer to assist solubilization of the cell wall material. The reaction mixtures were then adjusted to pH 6.5 by rapidly transferring the mixtures directly into an ammonium hydroxide solution (20% v/v) pre-cooled to -20°C. The solutions were then dialyzed at 4°C vs. deionized water for 3 d using 1,000 MWCO dialysis tubing (Spectrapor). Dialysates were concentrated by centrifugal vacuum-evaporation then subjected to ultracentrifugation (100,000 x *g*; 4°C, 4 h) to remove HF-insoluble material, yielding a supernatant containing the solubilized HF-SCWPs (crude SCWP preparation). The HF-insoluble residue consisted of peptidoglycan fragments and protein (amino acids) as determined by GC-MS analysis of the heptafluorobutyrate esters [28] and the TMS-methyl glycosides.

Crude HF-SCWP was dissolved in deionized water, filtered (0.45 μm cellulose acetate) (Mettler-Toledo Rainin LLC, Oakland, CA, USA), then purified by size exclusion chromatography on Superose-12 FPLC column (using “AKTA System”, Amersham) in 50 mM ammonium acetate pH 7.8 (refer to Supporting Information **S12 Fig**). The eluent was monitored by UV absorbance (210 nm/260 nm/280 nm) and refractive index, and fractions containing the SCWP were identified by glycosyl analysis. The HF-SCWP fractions were dialyzed (MWCO 2,000 Da, Spectra/Por, Spectrum Labs, Rancho Dominguez, CA USA) against water, lyophilized and used for NMR, linkage, and immunological analyses. Typical yields were 5 mg of purified SCWP per 40 mg of cell wall material.

### Glycosyl chemical analyses

Glycosyl linkage analysis was performed according to a modification of the method of Ciucanu and Kerek [29] as described previously [12]. The resulting partially methylated alditol acetates (PMAA) were dissolved in dichloromethane and analyzed by GC-MS using a DB-1 or SP-2330 capillary column as described [12, 30]. The carbohydrate compositions of the SCWP and derived fractions were determined by preparing the TMS-methyl glycosides with GC-MS (electron impact) analysis [31, 32] using a 30-m DB-5 capillary column (J&W Scientific, (Agilent Technologies Inc.) Folsom, CA USA). The absolute configuration of glycosyl residues was determined by preparing the diastereomeric TMS-(α)-2-butyl glycoside derivatives [33], with GC-MS analysis on a DB-1 column with comparisons to authentic D-Gal, D-GlcNAc, and D-ManNAc derivatives.

### Reduction of the reducing-end residue of *Bc* SCWPs

Portions of the *Bc* CA and CI HF-SCWPs (3–4 mg) were dissolved in water (200 μl) and mixed with 0.5 mg sodium borodeuteride (NaBD_4_) in water (200 μl) and reduced for 1 h at room temp. The reaction was neutralized by adding methanol containing dilute acetic acid, then evaporated several times from methanol in a centrifugal vacuum-evaporator (Speed Vac, Thermo-Fisher Scientific), before dialyzing (1000 MWCO membrane) for 1 d vs. deionized water. The reduced-HF-SCWP (red-HF-SCWP) was rechromatographed on Superose-12, isolated as described above, and subjected to NMR and linkage analyses as for the native materials.

### Nuclear magnetic resonance analyses

^1^H spectra and all two-dimensional homo- and heteronuclear spectra of the great ape derived HF-SCWPs were recorded at 25°C on a Varian Inova 600 MHz or Varian 800 MHz spectrometer, each equipped with a 3-mm cryogenic probe, using Varian software (Varian Medical Systems, Palo Alto, CA, USA). The SCWPs released by HF treatment, and purified by Superose-12 chromatography were exchanged with D_2_O (99%, 3 repetitions) and dissolved in 200 μl D_2_O (“100%”) yielding clear solutions at approximately 7 mg/ml in 3 mm tubes. The ^1^H-spectra/axes were referenced to 2,2-dimethyl-2-silapentane-5-sulfonate sodium salt (0.1% DSS in D_2_0) at δ_H_ 0.00 ppm, yielding the HOD resonance at 4.78 ppm. Certain samples, available only in small amounts (e.g., *Ba* CDC684 HF-SCWP, 400 μg) were dissolved in 90 μl of D_2_O and analyzed in 3 mm Shigemi tubes (Shigemi, Inc) using the cryogenic probe. The ^13^C axis in 2D analyses was then referenced to the calibrated proton axis, using the Varian software command “setref1(‘c13)”, an accepted method which uses the referenced proton dimension to calculate the 2^nd^ dimension (^13^C) according to a mathematical relationship. This procedure yields most reproducible and consistent referencing in biomolecular NMR, where the natural abundance of ^13^C and the % of DSS is too low to allow reliable detection of the DSS carbon signal, see *e*.*g*., Wishart, D. S., Bigam, C. G., Yao, J., Abildgaard, F., Dyson, H. J., Oldfield, E., Markley, J. L., and Sykes, B. D., "^1^H, ^13^C and ^15^N Chemical Shift Referencing in Biomolecular NMR," *J*. *Biomol*. *NMR 6*, 135–140 (1995).

^1^H-^1^H COSY [34] data were recorded in the absolute value mode with a 6.10 ppm spectral width and a matrix size of 1024 x 4096 complex points with 8 scans per increment. ^1^H-^1^H zTOCSY [35] was recorded with a mixing time of 150 ms and a matrix of 400 x 4096 at 32 scans per increment. Nuclear Overhauser effect analysis [36] was performed by phase-sensitive ^1^H-^1^H NOESY experiments, collected with a 150 ms mixing time and matrix size identical to that used for zTOCSY, with 64 scans per increment. Carbon chemical shifts and carbon-proton one-bond correlations were determined with a gradient-selected ^1^H-^13^C HSQC [37] collected in the ^1^H-detection mode. The acquisition time was 0.2 s, and the matrix size was typically 256 x 2,000 complex points, at 88 to 144 scans/increment with multiplicity distinction for primary/secondary protons. The coupling constant (j1ch) was set to 150 Hz and the carbon spectral width was 110 ppm, transmitter offset 65 ppm. Gradient ^1^H-^13^C-H2BC spectra [38] were acquired with a data matrix of 1600 (^1^H) x 140 (^13^C) complex points with acquisition times of 0.2 s (^1^H) and 22 ms (^13^C) respectively. Phase-sensitive ^1^H-^13^C HMBC spectra were acquired with 256 x 2048 complex points at 96 to 144 scans/increment. The carbon and proton sweep widths were identical to HSQC values, and the acquisition time (t2) was 0.27 s. The HMBC transfer delay was set to 50 ms (10 Hz), and 1-bond J-filter was 150 Hz. Anomeric configurations of the glycosyl linkages were assigned from carbon-proton coupling constants (*J*_C1,H1_) measured for the native SCWP by ^1^H-^13^C HSQC analysis without ^13^C decoupling. The acquisition time was 0.6 s, collecting 128 scans per increment; the j1ch was set to 160 Hz and carbon sweep width was 80–120 ppm.

### Molecular modeling

Using the GLYCAM Webtool (39), and the associated Glycam06 force field parameters for carbohydrates [40] we generated several minimal-energy conformers for oligosaccharide structural segments for representative HF-SCWPs. Both wire-frame and space filling renditions of these SCWP segments are shown, as described in Results section.

### Conjugation of HF-SCWPs to protein carriers

To facilitate immobilization to polystyrene microtiter immunoassay plates and to make polyclonal antibody, the HF-SCWP was conjugated to either bovine serum albumin (BSA; Sigma, St. Louis, Mo) or keyhole limpet hemocyanin (KLH; Sigma) as described by Leoff *et al*., 2009 [14].

### Antibody preparation

Monoclonal antibody (mAb) EAII-6G6-2-3 specific for *Ba* [10] was obtained from T. Abshire at the United States Army Medical Research Institute for Infectious Diseases (Fort Detrick, MD). Polyclonal anti-Ames HF-SCWP-KLH antiserum was generated in rabbits. Briefly, each of two female (2.0–3.5 kg) New Zealand White rabbits (Myrtle’s Rabbitry, Thompson Station, TN) was inoculated intramuscularly at two sites in the dorsal hind quarters. Each rabbit was immunized at 0, 14, 28, and 42 d with a total of 500 μg (primary vaccination) or 250 μg (all other vaccinations) of the *Ba* Ames HF-SCWP-KLH conjugate (1.0 mL total volume using Sigma Adjuvant System (Sigma, St. Louis, MO)). Serum used in this study was collected 14 d after the final immunization. All animal work was conducted with CDC IACUC approvals.

### Immunosorbent assay analyses

Monoclonal (mAb) and polyclonal (pAb) immuno-analyses were done using HF-SCWP conjugated to BSA (Sigma, St. Louis, MO) in electrochemiluminescent (ECL) immunosorbent assays (MSD; Meso Scale Discovery, Gaithersburg, MD). Each SCWP-BSA conjugate was adjusted to a concentration of 2 μg/mL total carbohydrate content in 0.01 M PBS pH 7.4. 25 μL of the concentration adjusted SCWP-BSA conjugates and 25 μL of 1 μg/mL of BSA protein (negative control) were incubated at +4°C for 16–18 h. For use, plates were washed, aspirated, and 150 μL of blocker (0.01 M PBS pH 7.4, 0.5% Tween-20, 5% skim milk [BD, Franklin Lakes, NJ]) was added to each well (60 min; 20–25°C). Plates were washed three times with wash buffer (0.01 M PBS, pH 7.4, 0.1% Tween-20) on an automated plate washer (BioTek ELx405, Winooski, VT).

Monoclonal antibody EAII-6G6-2-3 was evaluated over a range of 93.6 μg/mL to 0.0731 μg/mL in the blocker buffer (60 min; 20–25°C). After washing, bound EAII-6G6-2-3 was detected with MSD sulfo-tagged goat anti-mouse IgM detection antibody at 2.5 μg/mL and incubated at room temperature for 1 h with shaking. The conjugate was detected using MSD Read Buffer and plates were analyzed using a MSD SECTOR imager for emission at 620 nm.

Rabbit pAb was detected with 2.5 μg/mL of sulfo-tagged goat anti-rabbit IgM (MSD, Gaithersburg, MD). To assess the reactivity of each HF-SCWP sample to polyclonal anti-Ames SCWP-KLH, antiserum was added in two fold serial dilutions starting at 1/100 for each strain to generate a full dilution curve. Response curves were fitted to a 4 parameter logistic (4-PL) nonlinear regression model for quantitative analysis [41].

The quantitative endpoints for serological analysis were the effective concentration of mAb EAII-6G6-2-3 generating a 50% response (EC_50_) and the effective dilution of rabbit pAb generating a 50% response (ED_50_). The EC_50_ and ED_50_ values were determined as the reciprocal of the dilution corresponding to the inflection point (‘c’ parameter) of the 4 parameter logistical (4-PL) model to fit a dose-response curve to the data as described [41]. Reportable values were the geometric mean (GM) ED_50_ and EC_50_ values from three independent experiments. GM reactivity for each of the individual strains were compared using Student’s t-test with a P = 0.05 level of significance. Low EC_50_ values and high ED_50_ values indicated a high level of reactivity against specific HF-SCWP.

## Results

### Nuclear magnetic resonance (NMR) spectroscopy of the HF-SCWPs from *Bc* great ape isolates and comparison to human infection-associated *Bc* HF-SCWPs

**Fig 2:**
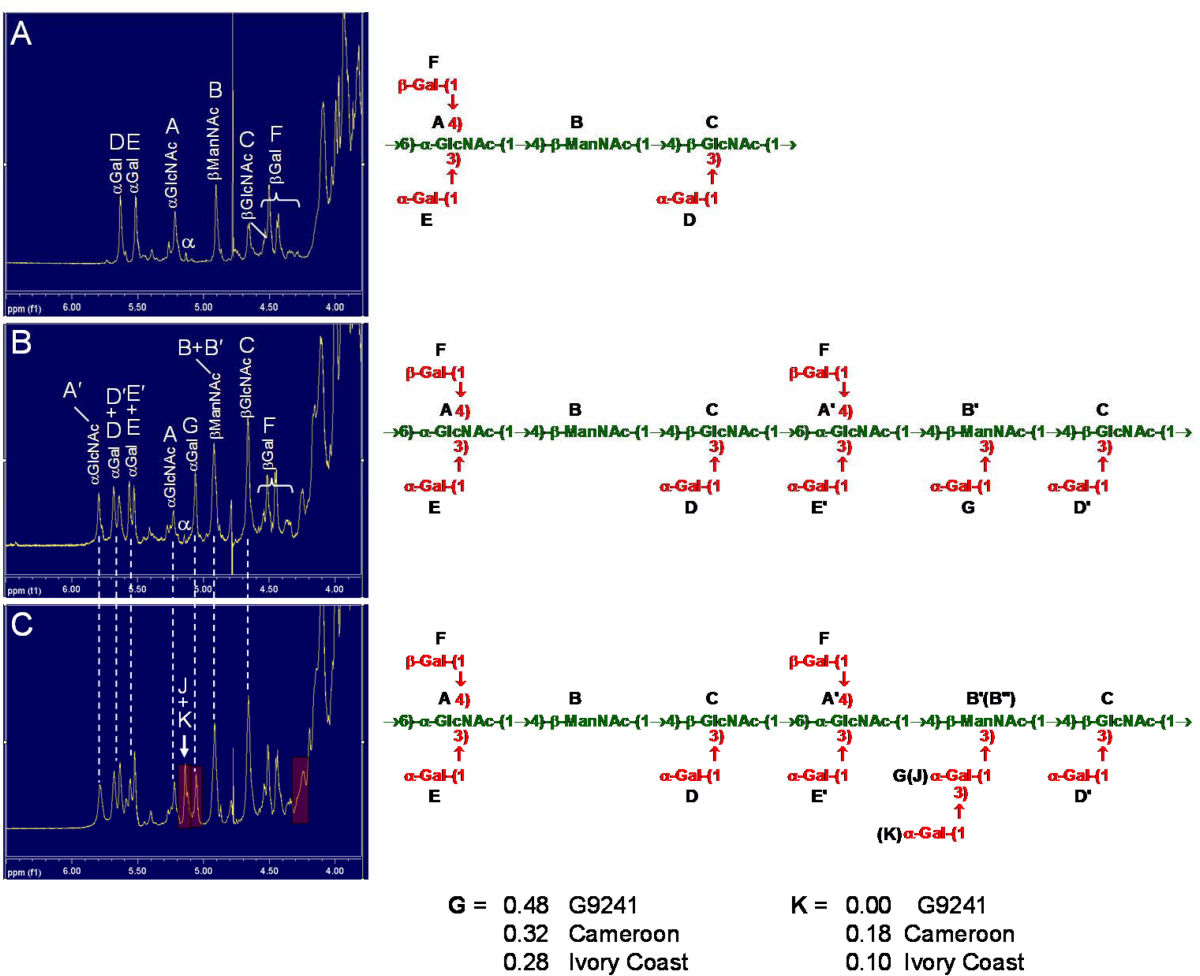
Comparison of the proton NMR spectra anomeric regions and repeating unit structures of HF-SCWPs from representative *B*. *anthracis* and related *B*. *cereus* strains. **(A)** *B*. *anthracis* Sterne 34F2; **(B)** the close phylogenetically related human infection-associated *B*. *cereus* strain G9241; **(C)** a great ape isolate, *B*. *cereus* strain CA (Cameroon). All SCWPs share a conserved HexNAc backbone; the *Ba* strains lack Gal substituents on the backbone ManNAc residue, while the examined human infection-associated *Bc* strains (G9241 and 03BB87) exhibit a Gal monosaccharide substitution (residue **G**) on a percentage of their ManNAc [27]. The great ape *Bc* isolates exhibit variable degrees of Gal monosaccharide and Gal disaccharide substitutions (residues **G**, **J,** and **K**) at ManNAc, which results in additional ManNAc signal heterogeneity (**B**, **B′**, and **B′′**). The repeating unit structures of the *Ba* and human infection–associated *Bc* HF-SCWPs *(panels A* and *B*), including glycosidic sequence and linkage positions, were deduced from ^1^H-^13^C HMBC, ^1^H-^1^H-NOESY, and chemical linkage analysis, previously published [12,26,27].

We have previously reported detailed NMR analysis of *Ba* derived SCWPs [12, 26], and human infection-associated *Bc* derived HF-SCWPs [27]. For comparison, the NMR parameters from one of the previously examined human infection-associated HF-SCWPs (from strain *Bc* G9241) are reported in **Table 2**, along with those from the great ape isolate *B*. *cereus* Cameroon (*Bc* CA). Our initial ^1^H-NMR analysis of the HF-SCWPs isolated from the *Bc* CA and *Bc* CI strains indicated that their structures were probably similar to those of the human infection-associated *Bc* G9241 and *Bc* 03BB87 strains, although with some interesting and significant differences in their ^1^H spectra (**Fig 2**). A number of points were noted in these proton spectra: (i) HF-SCWPs from both great ape isolates appeared to contain all of the NMR anomeric signals as found in the *Bc* G9241 HF-SCWP [27], however the ratios of these resonances were altered in both great ape isolates, compared to the *Bc* G9241; (ii) in addition to these “identical” components, both of the great ape isolates displayed two additional “new” signals in the anomeric region, not previously observed in the *Bc* G9241 and *Bc* 03BB87 human infection-associated strains. These new components were designated residues **J** and **K**, and their NMR parameters are listed in **Table 2**, along with the NMR parameters of all other repeating unit components.

**Table 2:** 600-MHz ^1^H and ^13^C NMR parameters observed for the HF-released secondary cell wall polysaccharide released by HF treatment of great ape isolate *B*. *cereus* strain CA cell walls^a^.

Compound Glycose Residue	(*J*_C1,H1_ Hz)	δH ppm					
		δ_*C*_ *ppm*^*b*^					
		1	2	3	4	5	6
***B*. *cereus* (CA) red-HF-SCWP** **A′ →**3,4,6)-α-D-Glc*p*NAc-(1**→**	**(179.5)**	5.77	4.15	3.98	4.06	3.86	4.11/4.11
		*96*.*46*	*52*.*77*	*75*.*32*	*76*.*96*	*69*.*88*	*68*.*02*
**A**^**c**^ **→3,4,6)-α-D-Glc*p*NAc-(1→**	**(177.0)**	5.23	4.08	4.01	4.03	3.95	4.12/4.12
		*99*.*08*	*53*.*43*	*75*.*56*	*76*.*96*	*71*.*47*	*67*.*28*
**B**^c^ **→**4)-β-D-Man*p*NAc-(1**→**	**(165.0)**	4.90	4.50	4.07	3.68–3.73	3.51	3.89/3.81
		*99*.*22*	*54*.*40*	*73*.*21*	*76*.*10*	*75*.*45*	*61*.*1*
**B′ →**3,4)-β-D-Man*p*NAc-(1**→**	**(165.1)**	4.91	4.65	4.24	4.09	3.52	3.89/3.75^d^
		*99*.*45*	*54*.*29*	*80*.*09*	*77*.*26*	*75*.*45*	*60*.*6*
**B′′ →**3,4)-β-D-Man*p*NAc-(1**→**	**(165.1)**	4.91	4.66	4.27	4.10	3.53	3.91/3.76^d^
		*99*.*45*	*54*.*29*	*80*.*09*	*77*.*26*	*75*.*55*	*60*.*6*
**C →**3,4)-β-D-Glc*p*NAc-(1**→**	**(159.4)**	4.65	3.91	3.91	4.10	3.52	3.92/3.71^d^
		*101*.*24*	*54*.*94*	*75*.*80*	*77*.*28*	*75*.*90*	*60*.*7*
**D α**-D-Gal*p*-(1**→**	**(178.5)**	5.67	3.79	3.72	3.98	3.82	~3.71/3.71
		*97*.*84*	*69*.*34*	*70*.*08*	*69*.*61*	*71*.*57*	*61*.*27*
**D′ α**-D-Gal*p*-(1**→**	**(177.1)**	5.62	3.80	3.73	3.99	3.82	~3.71/3.71
		*98*.*22*	*69*.*34*	*70*.*08*	*69*.*61*	*71*.*57*	*61*.*27*
**E α**-D-Gal*p*-(1**→**	**(177.0)**	5.54	3.75	3.71	3.98	3.85	~3.73/3.73
		*99*.*31*	*69*.*54*	*70*.*08*	*69*.*65*	*71*.*68*	→
**E′ α**-D-Gal*p*-(1**→**	**(177.1)**	5.51	3.75	3.72	3.97	3.85	~3.73/3.73
		*99*.*53*	*69*.*54*	*70*.*08*	*69*.*65*	*71*.*68*	*61*.*44*
**F**^c^ **β**-D-Gal*p*-(1**→**	**(163.7)**	4.43	3.52	3.63	3.93	3.65	3.82/3.82
		*103*.*41*	*71*.*69*	*73*.*19*	*69*.*20*	*76*.*19*	*61*.*60*
**G α**-D-Gal*p*-(1**→**	**(173.0)**	5.05	3.76	3.66	3.93	4.15	3.73/3.73
		*101*.*35*	*68*.*63*	*70*.*02*	*69*.*91*	*72*.*06*	*62*.*32*
**J →**3)-**α**-D-Gal*p*-(1**→**	**(173.0)**	5.11	3.91	4.18	4.18	3.73	3.63/3.54
		*101*.*11*	*67*.*03*	*77*.*20*	*65*.*84*	*72*.*80*	*63*.*18*
**K α**-D-Gal*p*-(1**→**	**(172.0)**	5.13	3.85	3.92	4.01	4.09	3.73/3.73
		*95*.*33*	*68*.*90*	*68*.*73*	*71*.*43*	*71*.*52*	*61*.*6*
***B*. *cereus* G9241 HF-SCWP**^**e**^ **A′ →**3,4,6)-**α**-D-Glc*p*NAc-(1**→**	**(178.7)**	5.79	4.15	3.99	4.07	3.86	4.14/4.14
		*96*.*61*	*52*.*79*	*75*.*56*	*77*.*70*	*69*.*98*	*68*.*0*
**A**^c^ **→**3,4,6)-**α**-D-Glc*p*NAc-(1**→**	**(177.0)**	5.21	4.08	4.01	4.03	3.96	4.14/4.13
		*99*.*19*	*53*.*43*	*75*.*72*	*77*.*06*	*71*.*47*	*67*.*4*
**B**^c^ **→**4)-β-D-Man*p*NAc-(1**→**	**(165.0)**	4.90	4.50	4.05–4.10	3.67–3.73	3.51	3.91/3.82
		*100*.*04*	*54*.*51*	*73*.*41*	*76*.*20*	*75*.*35*	*61*.*20*
**B′ →**3,4)-β-D-Man*p*NAc-(1**→**	**(165.1)**	4.91	4.65	4.24	4.10	3.52	3.91/3.89
		*99*.*40*	*54*.*51*	*80*.*28*	*77*.*27*	*75*.*56*	*60*.*7*
**C →**3,4)-β-D-Glc*p*NAc-(1**→**	**(159.4)**	4.65	3.90–3.91	3.90	4.10	3.52	3.92/3.74
		*101*.*54*	*55*.*15*	*75*.*98*	*77*.*31*	*75*.*99*	*60*.*7*
**D α**-D-Gal*p*-(1**→**	**(178.7)**	5.68	3.80	3.71	3.98	3.81	~3.72/3.72
		*97*.*91*	*69*.*54*	*70*.*19*	*69*.*76*	*71*.*70*	*61*.*4*
**D′ α**-D-Gal*p*-(1**→**	**(177.3)**	5.63	3.80	3.72	3.99	3.81	~3.72/3.72
		*98*.*33*	*69*.*54*	*70*.*19*	*69*.*76*	*71*.*70*	*61*.*4*
**E α**-D-Gal*p*-(1**→**	**(177.0)**	5.55	3.76	3.72	3.99	3.83	~3.83/3.74
		*99*.*40*	*69*.*76*	*70*.*19*	*69*.*76*	*71*.*70*	*61*.*6*
**E′ α**-D-Gal*p*-(1**→**	**(177.1)**	5.52	3.76	3.71	3.98	3.86	~3.83/3.74
		*99*.*62*	*69*.*76*	*70*.*19*	*69*.*76*	*71*.*70*	*61*.*6*
**F**^c^ **β**-D-Gal*p*-(1**→**	**(163.7)**	4.44	3.54	3.64	3.94	3.66	3.82/3.75
		*103*.*48*	*71*.*70*	*73*.*30*	*69*.*33*	*76*.*21*	*61*.*8*
**G α**-D-Gal*p*-(1**→**	**(172.6)**	5.05	3.77	3.65	3.93	4.15–4.20	3.73/3.73
		*101*.*56*	*68*.*68*	*70*.*19*	*69*.*97*	*72*.*13*	*62*.*46*

The *Bc* strain CA HF-SCWP was purified by Superose chromatography, then reduced with borodeuteride (red-HF-SCWP) prior to NMR analysis. Chemical shifts for the HF-SCWP derived from *B*. *cereus* strain CI are essentially superimposable with those of CA. For comparison, the parameters for the human infection-associated *B*. *cereus* G9241 HF-SCWP are also recorded (previously published [27], from Forsberg *et al*., 2011).

Additional signals: **pyruvate**: C1, (C = O) δ 177.04, (C2) δ 100.70, (C3) CH_3_ δ 25.29, CH_3_ δ 1.55; ***N*-acetyl:** βGlcNAc, C = O δ 174.46, CH_3_ δ 22.93, CH_3_ δ 2.08; αGlcNAc, C = O δ 176.20, CH_3_ δ 20.99, CH_3_ δ 2.08; βManNAc, C = O δ 175.97, CH_3_ δ 22.93, CH_3_ δ 2.03; **α-reducing end**: →3,6,4)-α-D-Glc*p*NAc, H1/C1 δ 5.13/91.67 (*J*_C1,H1_ 171.1 Hz), H2/C2 δ 4.06/53.5, H3 δ 3.94, H4 δ 3.83, H5 δ 3.75, H6 not assigned; **β-reducing end**: H1/C1 δ 4.74/95.32, H2/C2 δ 3.79/56.12, H3/H4/H5 = δ 4.07/3.87/3.51; H6 not assigned.

^a^ 600 MHz spectra measured at 25°C in D_2_O relative to internal DSS (δ_H_ 0.00 ppm). The chemical shifts and other spectral parameters for strains *Bc* CA and CI are virtually identical, except for differences in signal area ratios.

^b^ carbon δ_C_ obtained from ^1^H-^13^C HSQC spectra; carbonyl δ_C_ from the HMBC spectra.

^c^ additional heterogeneity exists for residues **A** and **F**, for all strains, as described previously for *Bc* G9241 [27]. The major spin systems **A** and **A′** are listed above in **Table 2**; less abundant systems (**A′′**, **D′**, **F′**, **F′′** etc.) arise from non stoichiometric attachment of Gal residues. Heterogeneity at residue **B**, arises from nonstoichiometric substitution of **B** with residues **G**, or (**J** + **K)**, yielding **B′** and **B′′** respectively.

^d^ assignments may be interchanged

^e^ for comparison, the parameters for the *Bc* G9241 strain HF-SCWP are also recorded (previously published [27] from Forsberg *et al*., 2011).

### Comparison HF-SCWP from *Ba* strains and infection-associated *Bc* strains

All of the *Ba* strains examined to date, including *Ba* Ames, *Ba* Pasteur, *Ba* Sterne 34F2, *Ba* 7702, and *Ba* CDC684 [12, 26], as well as examined strains of human infection-associated *Bc* G9241 and *Bc* 03BB87 [27] share an identical SCWP glycosyl backbone which consists of a linear HexNAc trisaccharide repeating unit: →4)-β-D-Man*p*NAc-(1→4)-β-D-Glc*p*NAc-(1→6)-α-D-Glc*p*NAc-(1→, designated here as: →4)**B**β(1→4)**C**β(1→6)**A**α(1→ to conform with previous nomenclature (26). With the exception of *Ba* strain CDC684, these polysaccharide backbones are highly galactosylated with α- and β-Gal residues, and the galactosylation pattern (location, linkage, anomeric configuration) is species/strain specific. HF-SCWPs from all examined *Ba* strains share the same overall galactosylation pattern yielding the hexasaccharide repeating unit shown in **Fig 2A**. Thus, the ratio of Gal substitution to HexNAc backbone residues is 1:1, which represents a high density of side chain attachment for a polysaccharide. In these *Ba* derived HF-SCWPs, a small percentage of the repeating units typically lack one or more of the Gal substituents, contributing to signal heterogeneity in their NMR spectra. This heterogeneity in Gal side residue attachment is observed in all the examined *Ba* and *Bc* derived HF-SCWPs.

In the human infection-associated *Bc* derived HF-SCWPs, a single additional Gal substitution is introduced at 50% of the backbone ManNAc residues, yielding the repeating structure and spectrum shown in **Fig 2B**. These *Bc* derived HF-SCWPs essentially consist of two types of repeating units, one *with*, and one *without* the Gal-substituted ManNAc. The introduction of this additional Gal residue modifies the magnetic environment of adjacent residues, such that their anomeric signals split, yielding twin signals, where one member of each pair arises from repeats which *lack* the Gal-ManNAc, and the other member signal arises from repeats which *carry* the Gal-substituted ManNAc [27]. Other ring positions are affected to lesser extents. The additional Gal attachment on these *Bc* derived HF-SCWPs results in polysaccharides having a side residue/backbone ratio of > 1:1. An extensive NMR comparison of the *Ba*-derived and human infection-associated *Bc*-derived HF-SCWPs was previously described [27] and representative HSQC spectra are included here for reference (Supporting Information **S1 Fig**).

In our present study, the NMR anomeric region of the great ape derived *Bc* strain CA HF-SCWP is compared with *Ba* and human infection-associated *Bc* derived HF-SCWPs in **Fig 2C**. The “new” signals are indicated, and their associated spin systems and residue identification are described in the following sections. Signals originally appearing in the *Ba* samples are again split into “twin signals”, but the splitting ratio is not 50/50, reflecting the fact that the Gal-substitution on ManNAc is not 50% in the great ape-derived SCWPs. This feature, a lower percentage Gal-substitution at the backbone ManNAc residue, is observed to an even greater degree with the great ape *Bc* strain CI HF-SCWP. The ^1^H-anomeric regions of *Bc* CI, *Bc* CA, the human infection-associated strain *Bc* G9241, and *Ba* Sterne 34F2 are all compared in **S2 Fig**, demonstrating their relationship and differences. The ^1^H-^1^H TOCSY spectra of the great ape *Bc* CA HF-SCWP and the previously analyzed human infection-associated *Bc* G9241 HF-SCWP are compared in **S3 Fig**, demonstrating the similarity of the conserved residues, and the presence of new glycosyl systems in the great ape isolate. The ^1^H-^1^H TOCSY spectra of SCWPs from the two great ape isolates *Bc* CA and *Bc* CI are compared in **S4 Fig** demonstrating their overall similarity.

### Identification of new Gal substituents on SCWPs of great ape strains *Bc* CA and *Bc* CI

**Fig 3:**
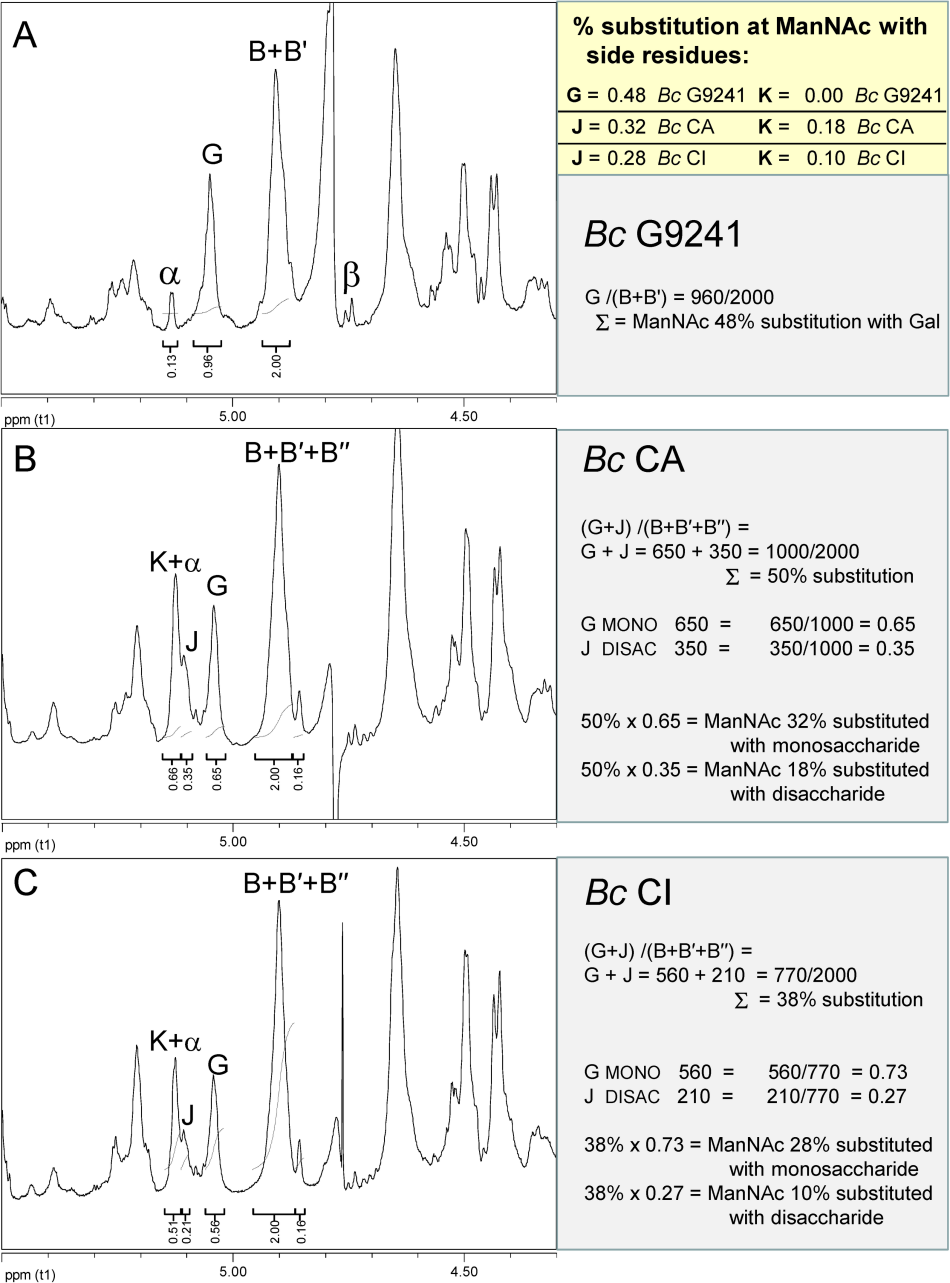
Expanded region of proton NMR spectra identifying the locations and area ratios of the Gal substituents on HF-SCWPs from *B*. *cereus* strains. **(A)** human isolate *B*. *cereus* G9241; **(B)** great ape isolate *B*. *cereus* CA (Cameroon); **(C)** great ape isolate *Bc* CI (Côte d’Ivoire). The α-anomeric signal originates from the free reducing end of all these polysaccharides, and co-migrates with a new residue (**K**) in the *Bc* CA and *Bc* CI SCWPs. This reducing end (α-GlcNAc residue) was removed by borodeuteride reduction for subsequent experiments, to facilitate integration and characterization of the residue **K** system. The percentage of Gal (**G**) and Gal-Gal disaccharide (**J**+**K**) substitution at ManNAc residues (**B**+**B′**+**B″**) is estimated by examination of the signal areas for these residues. The residue **G** spin system undergoes a shift at several positions, and is designated residue **J**, when it is substituted by residue **K** (refer to **Table 2**).

In **Fig 3**, we compare the regions of the ^1^H-NMR spectra where the new, additional signals (**J** and **K**) are observed in the great ape derived HF-SCWPs compared to *Bc* G9241. This region also reveals the percentage of ManNAc residues (**B′**) that are substituted with an α-Gal residue (**G**), originally identified in the human infection-associated *Bc* G9241 HF-SCWP [27]. In **Fig 3A**, the HF-SCWP from *Bc* G9241 demonstrates the near 50% substitution of the ManNAc residue (**B** +**B′**) with this Gal(α1→3) substituent (**G**). In comparison, the HF-SCWPs from both *Bc* strains CA and CI (**Fig 3B and 3C**) appeared to show a lowered ratio of ManNAc substitution by residue **G**, as calculated by integration of the anomeric signals for residues **G**, **J**, and (**B**+**B′**+**B′′**). Thus, area integration suggested that substitution of ManNAc by **G** occurs at a lower percentage in the great ape derived SCWPs, compared to the *Bc* G9241.

**Fig 4:**
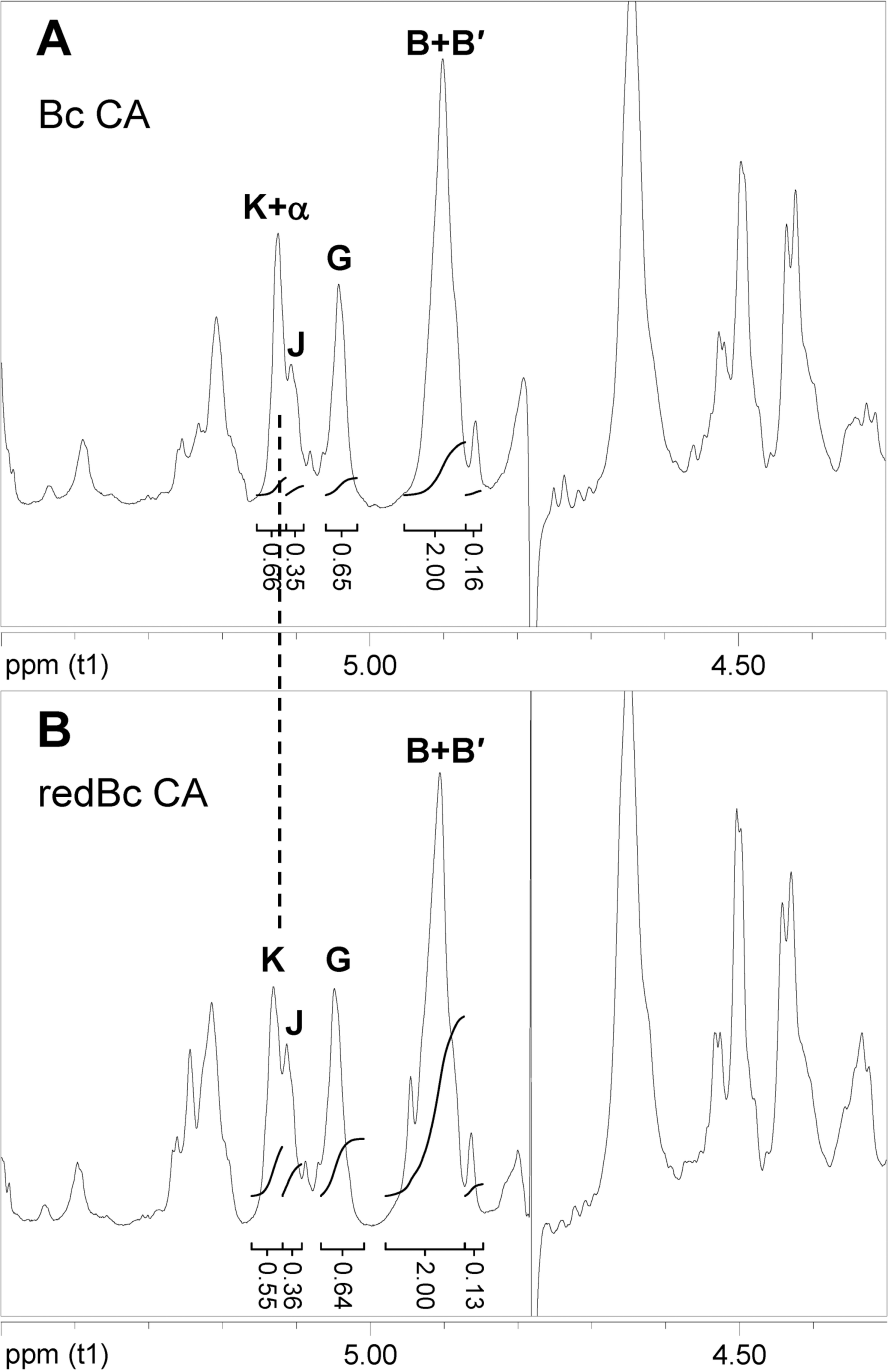
Proton NMR spectra comparing the *Bc* Cameroon HF-SCWP before and after reduction of the reducing end with borodeuteride. The effect of borodeuteride reduction on the signal at (**K** + α) is demonstrated: **(A)** native *Bc* CA HF-SCWP before reduction; **(B)** the *Bc* CA HF-SCWP after reduction of the reducing end (red-HF-SCWP). The contribution from the α-reducing end anomeric signal to residue **K** signal area was almost completely eliminated by 1 h treatment, allowing unambiguous assignment of residue **K** system and area measurements. Following reduction, the **K**/**J** ratio appears to approach 1:1, reflecting the presence of the **K**α(1→3)**J**α(1→ disaccharide. Considerable overlap of the **K** and **J** anomeric signals appears to result in some inaccuracy in the integration, as the HSQC spectra demonstrate that both α- and β- reducing end anomeric protons were absent following borodeuteride reduction (see **S4 Fig**).

We also initially found that one of the new anomeric signals (assigned residue **K**, H1 δ_H_ 5.13), actually had the same chemical shift as a low-abundance resonance previously observed in all *Ba* and *Bc* HF-SCWPs, and identified as the free reducing end (GlcNAc, α-anomer) of the polysaccharides [27]. The presence of this free α-reducing end thus interfered with area integration and spectral characterization of the new residue **K** in the great ape isolates. To remove this interference, the *Bc* CA and *Bc* CI HF-SCWPs were reduced at the reducing end with sodium borodeuteride, yielding the reduced-HF-SCWPs (red-HF-SCWP). In **Fig 4**, the effect of reduction on the α-reducing end signal is indicated by a decrease in the relative signal area at **K+α**, in the *Bc* CA red-HF-SCWP. Although reduction was incomplete, the area of **K+α** was decreased substantially to the point where it was roughly equivalent to the area of residue **J**, due to removal of mostthe α-reducing end contribution. To further clarify the effect of reduction on the SCWPs, the ^1^H-^13^C-HSQC spectra of the anomeric regions, before and after reduction, were examined (**S5 Fig**). These HSQC spectra demonstrate that the NaBD_4_ treatment was effective in removing both the α- and β- reducing end anomeric signals and it did not appear to alter the repeating anomeric signals of the HF-SCWPs in any manner. A comparison of the TOCSY spectra of this region before and after reduction is also provided (**S6 Fig**). Having removed interference by the α-reducing end, the identification and location of the new residues **K** and **J** in the red-HF-SCWP samples were investigated.

**Fig 5:**
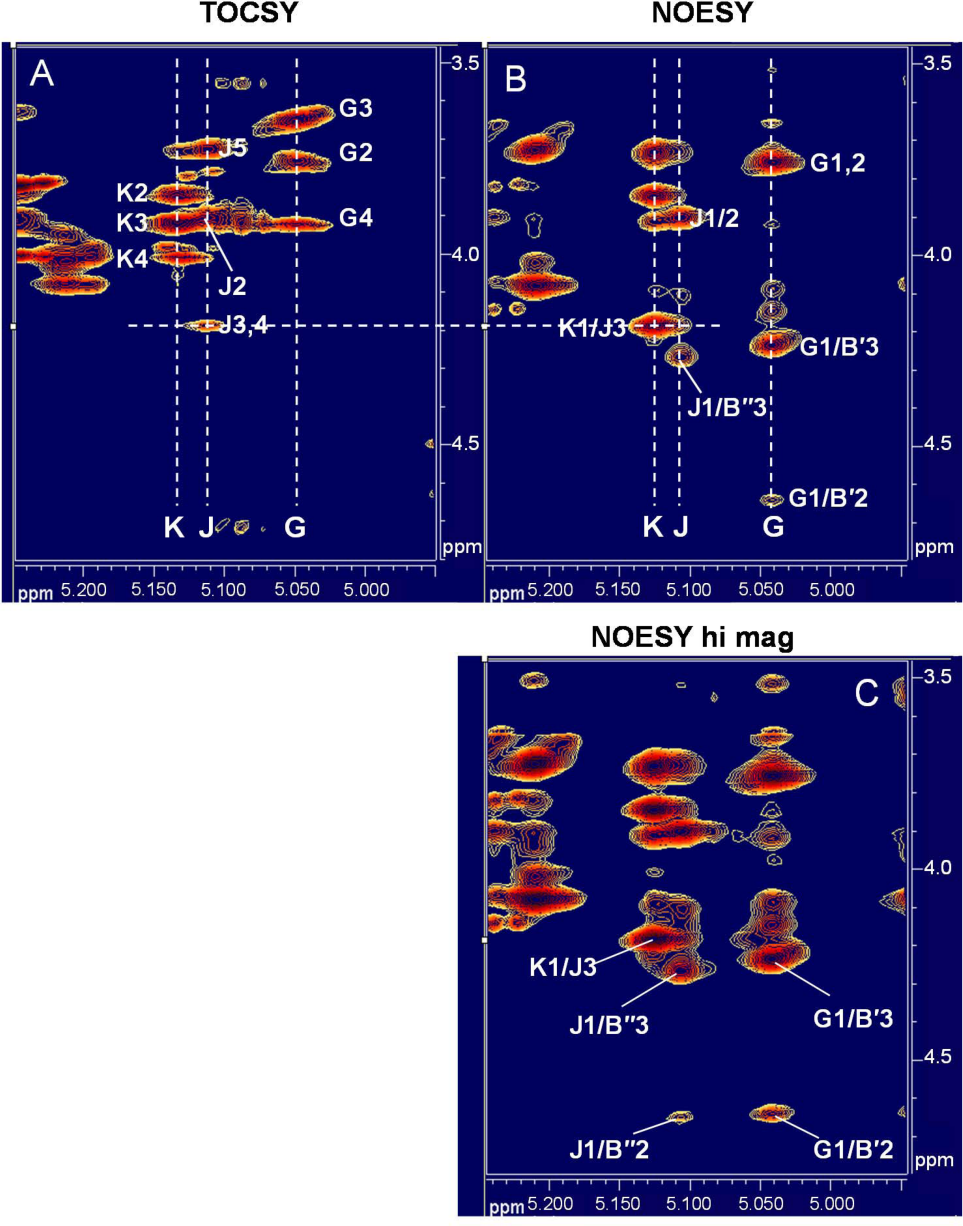
Partial ^1^H-^1^H TOCSY and ^1^H-^1^H NOESY spectra defining the features and glycosidic linkages of residues K, J, and G in the *borodeuteride-reduced* HF-SCWP from *Bc* CA. **(A)** TOCSY, showing *intra*-residue connectivities for residues **K**, **J**, and **G**; **(B)** NOESY, showing *inter*-residue NOEs for the **K**1→**J**3, **J**1→**B″**3, and **G**1→**B′**3 glycosidic linkages; **(C)** NOESY at higher intensity, revealing weaker NOEs resulting from the close proximity of **J**1 and **G**1 to the 2-position proton of residues **B″** and **B′**. The δ_H_/δ_C_ of **B″**3, **B′**3, **B′**2, and **B″**2 are unique in the HSQC spectra and key indicators of linkage position for the structural features unique to the great ape SCWP isolates (**Table 2**and **S7 Fig**). In the TOCSY (*panel A*) the connectivity “**J**3,4” designates correlations between **J**1/**J**3, and between **J**1/**J**4; the **J** protons H3 and H4 have identical δ_H_ (**Table 2**).

The *J*_C1,H1_ coupling constants for residues **J** and **K** were 173.0 Hz and 172.0 Hz (**Table 2**), indicating that both residues were probably of α-anomeric form. ^1^H-^1^H-COSY and ^1^H-^13^C HSQC analysis identified H2 for each residue, indicating that neither was a 2-amino sugar. ^1^H-^1^H COSY, and TOCSY analysis (**Fig 5A**) revealed weak magnetic transfer beyond position 4 for both residues, consistent with the *galacto*-configuration. New, hydroxy-methylene protons, not observed in the *Bc* G9241 spectrum, were identified in the HSQC spectra of *Bc* CA (and *Bc* CI) for residue **J** at δ 3.63/3.54/63.18 (H6/C6, **S7 Fig**), and TOCSY analysis demonstrated connectivity to **J** H5/C5 (δ_H_/δ_C_ 3.73/72.80, another new signal not observed in the *Bc* G9241 HSQC), and subsequently to H4/C4 (δ_H_/δ_C_ 4.18/65.84) of residue **J** (**Table 2**and **S7 Fig**). Additional HSQC and TOCSY spectra comparing relevant regions from the great ape *Bc* CA and human infection-associated *Bc* G9241 HF-SCWPs are shown in **S8**and **S3 Figs**.

The **K** system was defined with the assistance of HSQC and H2BC one and two-bond couplings for assignment of positions 6 and 5. H2BC revealed methylene protons (δ_H_ 3.73/3.73) which coupled to C6 (δ_C_ 61.6) and C5 (δ_C_ 71.52), with assignment of H5 (δ_H_ 4.09) from a new HSQC signal, not present in the Bc G9241 spectrum. Partial TOCSY spectra defining residues **J** and **K** are shown in **Fig 5A**. The *galacto*-configuration was assigned to both **J** and **K** on the basis of weak scalar coupling at H4, the relative downfield shift at H4, and intra-residue NOEs between H3-H4 and H5-H4 in addition to H3-H5. As described below, GC-MS analysis of the TMS-methyl glycosides and partially methylated alditol acetates detected elevated levels of Gal in the great ape HF-SCWPs, compared to the *Bc* G9241, and failed to detect any other “new” hexoses.

### Location of additional α-Gal substituents (residues J and K) on the great ape isolates *Bc* CA and *Bc* CI HF-SCWPs

In the SCWPs from human infection-associated strains *Bc* G9241 and *Bc* 03BB87, the attachment of the α-Gal (here assigned residue **G**) to the 3-position of 50% of the ManNAc residues was clearly demonstrated by inter-residue NOEs and trans-glycosidic HMBC connectivities [27]. This substitution of ManNAc at the 3-position results in a unique signature at ManNAc **B′** H3/C3 (δ_H_/δ_C_ 4.24/80.28) which differs from that of the unsubstituted ManNAc residue **B** (**Table 2**), and is unique in the HSQC spectrum. For both great ape derived HF-SCWPs, a certain percentage of the backbone ManNAc residues were found to be substituted at O3 by an α-Gal residue (**G**) (compare residues **B** and **B′**, the latter having H3/C3 δ_H_/δ_C_ 4.24/80.09, **Table 2**). Inspection of the ^1^H-^1^H NOESY spectrum for *Bc* CA red-HF-SCWP revealed an inter-residue NOE between **G**1 (δ_H_ 5.05) and **B**′3 (δ_H_ 4.24), **Fig 5B**, indicating that Gal is linked α1→3 to ManNAc in these great ape isolates as well. Most important, further inspection of the NOESY spectra indicated that the new residue **J** anomeric proton (δ_H_ 5.13) showed only two strong NOEs, to a proton at δ_H_ 3.91 (identified as H2 from COSY) and an NOE to a new proton at δ_H_ 4.27 (**Fig 5B**), subsequently identified as H3 of a new variant of the ManNAc residue (assigned **B′′**). These results indicated that the α-Gal residue **J** is also linked to the 3-position of a percentage of ManNAc residues, however, the residue **J** spin system differed from that of residue **G**. Inspection of the **J** spin system defined above suggested that it may itself be substituted at H3/C3 (δ_H_/δ_C_ 4.18/77.20) by an unknown residue. This substituent was readily identified as the new α-Gal residue **K**, having a strong inter-residue NOE between **K**1 (δ_H_ 5.13) and **J**3 (δ_H_ 4.18), **Fig 5B and 5C,** and NOEs to protons **J**2 (δ_H_ 3.91) and (intra-residue) to **K**2 (δ_H_ 3.85). The resulting glycosidic sequence, **K**α1→3**J**α1→3**B′′**, was substantiated by a trans-glycosidic ^1^H-^13^C HMBC connectivity between **J**H1 (δ_H_ 5.11) and **B′′**C3 (δ_C_ 80.09) (shown in **S9 Fig**). The linkage **G**α(1→3)**B′** was also confirmed by a trans-glycosidic HMBC between **G**H1 (δ_H_ 5.05) and **B′**C3 (δ_C_ 80.09), as previously observed for the human infection-associated *Bc* strains G9241 and 03BB87. Additional confirmation for intra-residue assignments defining the new residues in the *Bc* CA red-HF-SCWP were confirmed by 2-bond ^1^H-^13^C H2BC connectivities observed for **J**H1/**J**C2 (δ_H_ 5.11/δ_C_ 67.03); **K**H1/**K**C2 (δ_H_ 5.13/δ_C_ 68.90), and **K**H2/**K**C1 (δ_H_ 3.85/δ_C_ 95.33) *inter alia*. shown in **S10 Fig**.

To further clarify the nature of the new residues and their linkages, chemical linkage (methylation) analysis was performed and the resulting PMAA derivatives were compared for great ape derived HF-SCWPs and the previously analyzed human infection-associated *Bc* G9241 HF-SCWP. Only the great ape derived HF-SCWP yielded low levels of a 3-linked Gal PMAA derivative, not previously observed with any of the human pathogen derived strains (**S11 Fig**). Together these results indicated that HF-SCWPs from great ape derived CA and CI strains exhibit a nearly identical structure as that from *Bc* G9241, with one interesting and rather unusual difference. All contained a portion of their backbone ManNAc residues substituted with α-Gal at the 3-position (a feature that is lacking in the *Ba* derived HF-SCWPs). However, the great ape isolates exhibited a further modification, in which a percentage of the ManNAc residues carried a Galα1→3Galα1→3 disaccharide substituent. **Table 3**summarizes the relative percentage of these substituents, estimated from the NMR signal areas of anomeric resonances (**Fig 3**), for these human infection-associated and great ape-isolated *Bc* strains.

**Table 3:** Percent and type of structural substituent at the 3-position of backbone ManNAc residues in HF-SCWPs from examined infection-associated *B*. *cereus* strains.

Strain	Percent^*a*^ and Type of Substitution at ManNAc^*b*^		Occurrence^*c*^
*Bc* G9241	50%	Galα(1→3)	5/10 RUs
*Bc* 03BB87	50%	Galα(1→3)	5/10 RUs
*Bc* CA	32%	Galα(1→3)	3.2/10 RUs
	18%	Galα(1→3)Galα(1→3)	1.8/10 RUs
*Bc* CI	28%	Galα(1→3)	2.8/10 RUs
	10%	Galα(1→3)Galα(1→3)	1.0/10 RUs

The isolated HF-SCWPs from *B*. *cereus* G9241, *Bc* 03BB87, *Bc* Cameroon (CA) and *Bc* Côte d’Ivoire (CI) strains all contain on average 11 repeating units, consisting of a HexNAc trisaccharide backbone and its attached Gal side residues. This conclusion is based on two independent methods: integration of NMR anomeric signal areas (repeating signals compared to reducing end signals; and on the calculated MW as estimated by SEC chromatography on Superose, see **S12 Fig**). The structural feature that distinguishes the *Bc* derived SCWPs from each other, and from *B*. *anthracis* derived SCWPs, is that these pathogenic *Bc* strains contain variable amounts of Gal or Gal→Gal disaccharide substituent at the backbone ManNAc residue in a percentage of their repeating units; all examined *Ba* derived SCWP lack this substituent.

^*a*^ estimated from integration of NMR anomeric signals from the free-reducing end and the repeating unit residues in each HF-SCWP, and confirmed by Kav values obtained by SEC (**S12 Fig**).

^*b*^ all of these substituents at ManNAc are at the 3-position

^*c*^ expressed on the basis of “per 10 RU” (repeating units)

### SCWP reactivity with polyclonal anti-Ames SCWP-KLH antibody

**Fig 6:**
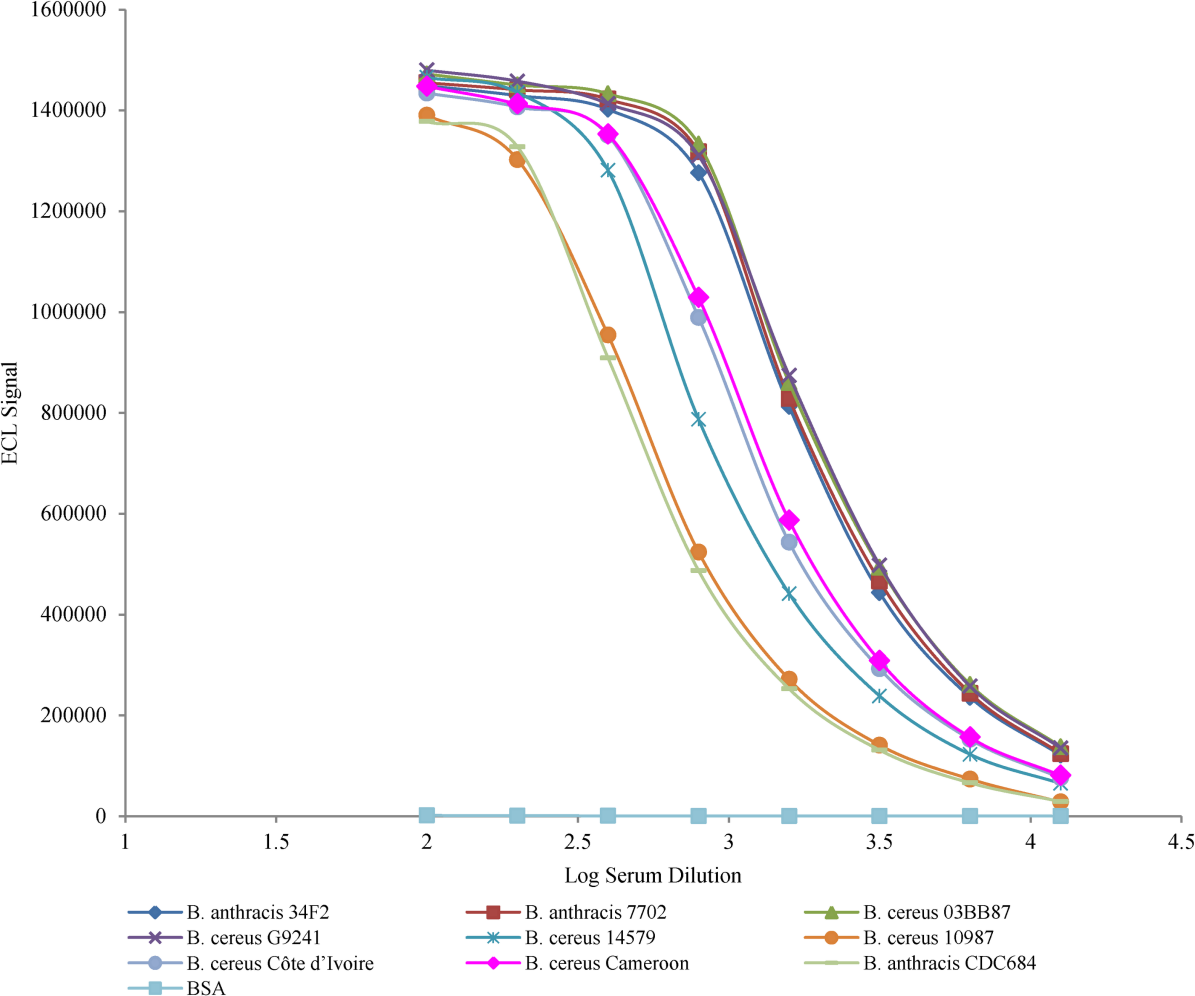
Reactivity of polyclonal antibody anti-Ames HF-SCWP-KLH with HF-SCWPs from *Bc* group strains using an ECL immunosorbent assay. Meso Scale Discovery (MSD) multi-array high bind 96 well plates were coated with a fixed concentration of SCWP antigen (2 μg/ml) from different *Bacillus* species and probed with the polyclonal antibody in serial two fold dilutions. Bound anti-Ames HF-SCWP-KLH antibody was detected by 2.5 μg/ml of sulfo-tagged goat anti-rabbit IgM detection antibody. Plates were measured in the SECTOR imager where a voltage is applied to the plate electrodes and light at 620 nm measured. Data points are the average of three independent experiments. Error bars represent one standard error. Reactivity reported as the effective dilution (ED_50_) titer, midpoint of the curve, in **Table 4**.

For comparative analysis, strains were grouped based on their structural similarity in the aminoglycosyl backbone and its galactosyl modifications (**Table 4**). The HF-SCWPs from all examined isolates reacted with the polyclonal (pAb) anti-Ames HF-SCWP antiserum. The highest reactivity was observed with the HF-SCWPs from strains *Ba* Sterne 34F2 and *Ba* 7702 (**Fig 6, Table 4**). There were no significant differences in the reactivity of the pAb serum with HF-SCWPs from *Ba* Sterne 34F2, *Ba* 7702 and infection-associated *Bc* 03BB87 and *Bc* G9241 strains (p = 0.17). There was, however, an approximately 35% reduction in pAb binding to the HF-SCWPs from the great ape disease causing strains *Bc* CI and *Bc* CA compared to the infection-associated *Ba* and *Bc* strains (p <0.05). There was an approximately 70% reduction in pAb binding to the HF-SCWP from the avirulent *Ba* CDC684 strain and the non-infection-associated strain *Bc* ATCC 10987 as well as an approximately 50% reduction of pAb binding to the HF-SCWP from strain *Bc* ATCC 14579 when compared to the infection-associated *Ba* and *Bc* strains (p <0.05). The ED_50_ values obtained with HF-SCWPs from the two non-infection-associated strains *Bc* ATCC 14579 and 10987 were statistically different from each other (p <0.05). However, while there was a significant difference between the HF-SCWP reactivities from the non-infection- associated type strain *Bc* ATCC 14579 and *Ba* CDC684 (p <0.05), there was no significant reactivity difference between dairy isolate *Bc ATCC* 10987 and the highly attenuated, Gal-substitution negative *Ba* CDC684 strain (p = 0.24).

**Table 4:** Immunochemical analysis of HF-SCWP with monoclonal and polyclonal antisera.

**A ED**_**50**_ **and EC**_**50**_ **values obtained from HF-SCWP isolated from different *B*. *anthracis* and *B*. *cereus* strains**											
**HF-SCWP Structure**				**Structural Group Assignment**		**SCWP Strain Origin**		**ED**_**50**_ **of polyclonal anti-Ames HF-SCWP-KLH (pAb) Antiserum**		**Monoclonal** **EAII-6G6** **Reactivity EC**_**50**_ **(**μ**g/mL)**	
**Refer to Fig 1 for structures of HF-SCWP**				1		*Ba* Sterne 34F2 *Ba* 7702		1745.78 1786.23		0.56 0.56	
				2		*Bc* G9241 *Bc* 03BB87		1877.99 1843.57		13.60 7.22	
				3		*Bc* CI *Bc* CA		1143.13 1211.98		49.94 64.32	
				4		*Bc* ATCC10987		546.59		141.89	
				5		*Bc* ATCC14579		842.40		223.45	
				6		*Ba* CDC684		517.16		356.04	
**B p-Values of pairwise antibody reactivity comparison.**											
**Structural Group**		**1**	**2**		**3**		**4**		**5**		**6**
**Monoclonal Antibody Reactivity Comparisons**	**1**	**-**	0.17		< .0001		< .0001		< .0001		< .0001
	**2**	0.0035	-		< .0001		< .0001		< .0001		< .0001
	**3**	0.0014	0.0043		-		< .0001		0.0014		< .0001
	**4**	0.0036	0.0050		0.037		-		0.0044		0.24
	**5**	0.00074	0.00098		0.0072		0.30		-		0.0029
	**6**	0.033	0.035		0.045		0.18		0.21		**-**

For the strains grouped together in structural group 1, 2, or 3, both the ED_50_ (1) and the EC_50_ (2) derived p-values showed that the differences in reactivity obtained with HF-SCWP-specific pAb and mAb EAII-6G6 and respective SCWPs are not statistically significantly different. (1) ED_50_ -derived p-values: *Ba* Sterne 34F2 vs. *Ba* 7702**,** p-value 0.40; *Bc* 03BB87 vs. *Bc* G9241, p-value 0.42; *Bc* CI vs. *Bc* CA; p-value 0.23. (2) EC_50_-derived p-values: *Ba* Sterne 34F2 vs. *Ba* 7702, p-value 0.48**;** *Bc* 03BB87 vs. *Bc* G9241, p-value 0.13; *Bc* CI vs. *Bc* CA, p-value 0.31.

ED_50_ titer (effective dilution giving 50% of maximum response) was determined as the reciprocal of the dilution of the polyclonal antibody corresponding to the inflection point of the antibody dose response curve using a 4-PL model [41]. EC_50_ (mAb concentration effecting 50% of maximum response) was determined as the concentration of monoclonal antibody corresponding to the inflection point of the antibody dose response curve using a 4-PL model. High ED_50_ values and low EC_50_ values indicated a high level of reactivity against specific HF-SCWPs.

The HF-SCWP pAb reactivities separated into distinct groups (**Table 4**). HF-SCWPs from the strains *Ba* Sterne 34F2 and *Ba* 7702 (Structural Group 1 ED_50_ values: 1746 and 1786, respectively; p = 0.4) had similar immuno-reactivities to the human infection-associated strains *Bc* G9241 and *Bc* 03BB87 (Structural Group 2 ED_50_ values: 1844 and 1878, respectively; p = 0.42). The immuno-reactivities of these two groups were not significantly different from each other (p = 0.17, **Table 4**). HF-SCWPs from the *Bc* CI (ED_50_ value: 1143) and *Bc* CA (ED_50_ value = 1212) were not statistically different from each other (p = 0.32). The lowest reactivities were observed with the HF-SCWP from *Ba* CDC684 (ED_50_ value: 517), and from the non-infection-associated strains *Bc* ATCC 10987 and *Bc* ATCC 14579 (ED_50_ values: 547 and 842, respectively).

### HF-SCWP reactivity with monoclonal antibody EAII-6G6

**Fig 7:**
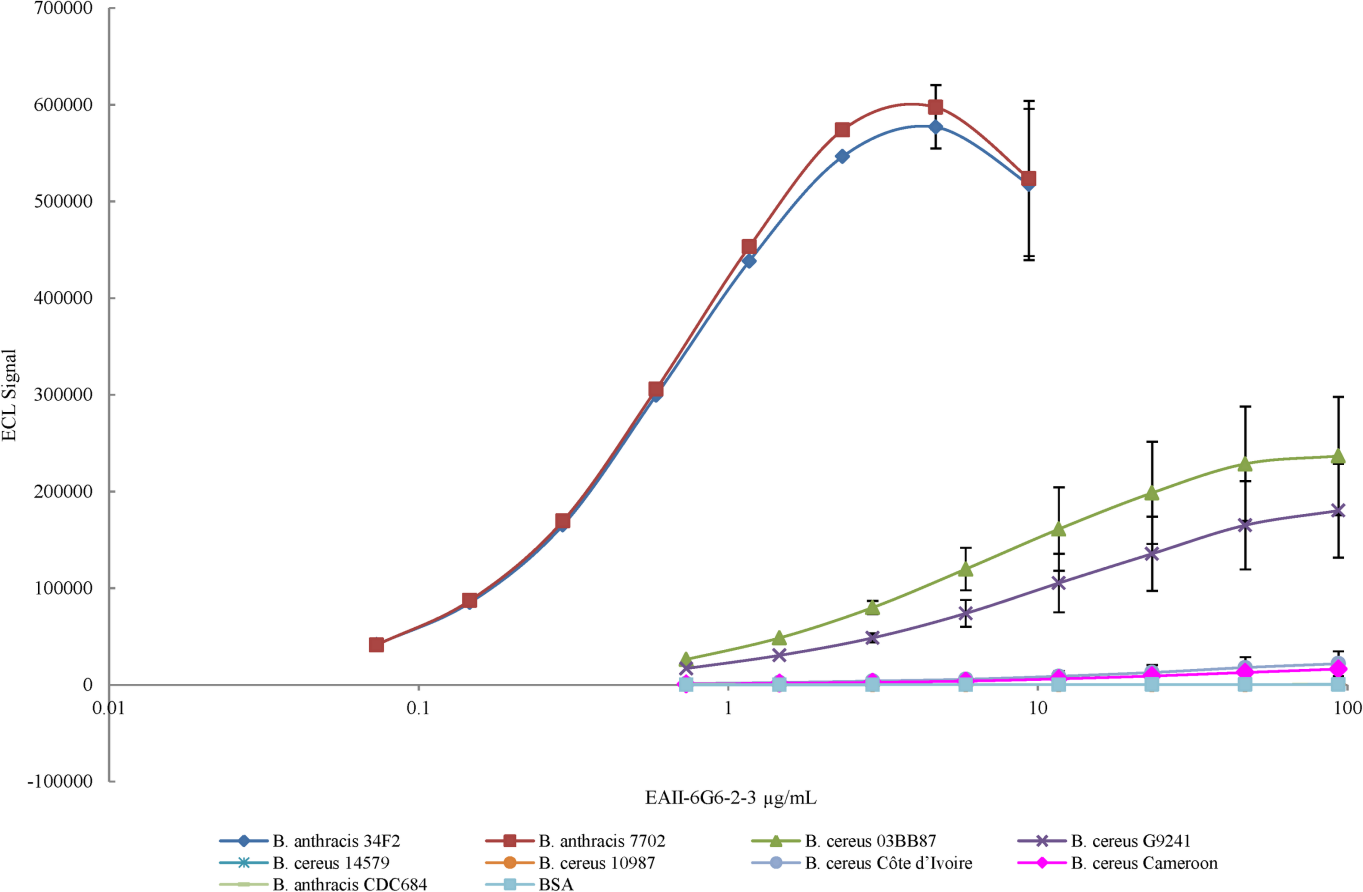
Reactivity of monoclonal antiserum EAII-6G6-2-3, specific for *Ba* neutral cell wall polysaccharide [19], with HF-SCWPs from *Bc* group strains. Meso Scale Discovery (MSD) multi-array high bind 96 well plates were coated with a fixed concentration of HF-SCWP antigen (2 μg/ml) from different *Bacillus* species and probed with the monoclonal antibody in serial two fold dilutions. Bound antibody was detected by using 2.5 μg/ml of sulfo-tagged goat anti-mouse IgM detection antibody. Data points are the average of three independent experiments. Error bars represent one standard error. Reactivity reported as effective concentration (EC_50_) titer in **Table 4**.

**Fig 8:**
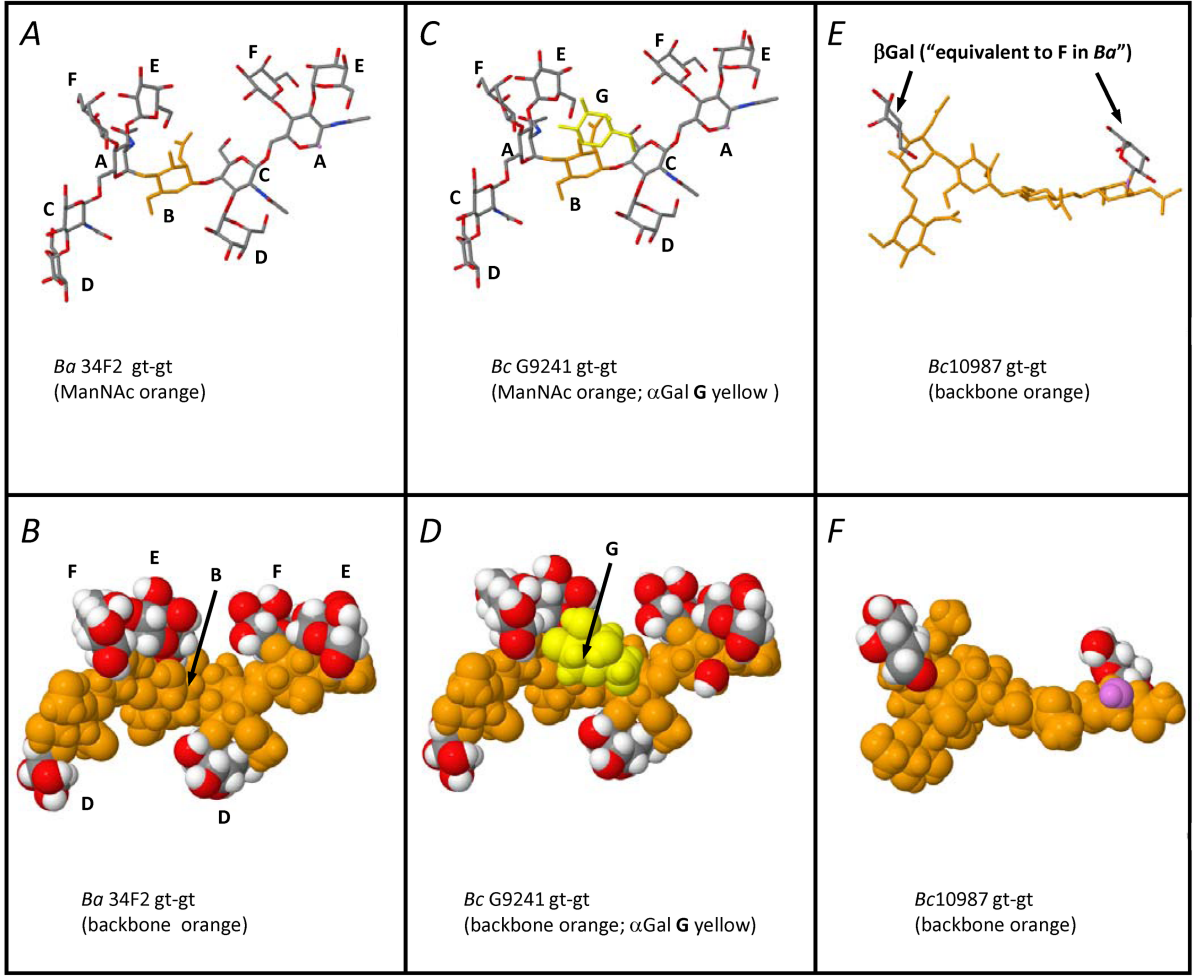
Comparison of minimal energy *gt-gt* conformers for repeating segments from the *Ba* Ames, *Bc* G9241, and *Bc* ATCC 10987 HF-SCWPs. *Top panels* (*A*, *C*, *and E*) are wire frame models, absent all hydrogens for clarity; (*bottom panels B*, *D*, *F*) show their corresponding space filling renditions, with all hydrogens. The space filling models depict Gals using standard CPK colors (gray = carbon; red = oxygen; white = hydrogen). To distinguish the HexNAc backbones, all backbone atoms are shown in orange. The *Ba* Ames segment (*panels A*, *B*) is an eleven residue segment depicting the backbone residue sequence **C-A-B-C-A** with its six Gal substituents (residues are labeled according to the scheme presented in **Figs 1 and 2**and **Table 2**). In the equivalent segment from the human infection-associated *Bc* G9241 model (*panels C*, *D*), the αGal substituent introduced at O3 of ManNAc (*shown in yellow*) is clearly noticeable. We propose this substituent could interfere with mAb recognition of the *conserved* Gal residues (particularly residues **E** and **F**), accounting for reduced mAb affinity for *Bc* G9241 (**Fig 7**). In the frame shift shown here (**C-A-B-C-A**) ManNAc is shown in the center, since structural alterations at ManNAc are the focal point responsible for the differences in EAII-6G6 mAb binding. Although not shown here, further attachment of additional Gal in the form of an (αGal1→3αGal) disaccharide at this location (residues **J** and **K**) is proposed to cause additional reduction of mAb binding (as observed with the great ape derived HF-SCWPs, **Table 4**). In *panels E and F*, an “equivalent” segment of the *Bc* ATCC 10987 SCWP is depicted; this SCWP consists of a different HexNAc backbone sequence than that shared by all the pathogenic strains, and with only a single βGal attachment. A labeling nomenclature for the individual glycosyl residues of this non-binding structure was not assigned, since the overall structure is not shared with any of the infection-associated SCWPs. The models demonstrate the striking difference in α/β-Gal spatial arrangement presented by the high-binding, intermediate binding, and non-mAb-binding SCWP. Models were generated using the Carbohydrate Builder Webtool [39] and the GLYCAM06 force field parameters [40].

There was a hierarchy of mAb reactivity (EC_50_ values) with the examined HF-SCWPs (**Table 4**). The HF-SCWPs from *Ba* Sterne 34F2 and *Ba* 7702 strains had the highest reactivities (EC_50_ values of 0.56 and 0.56 μg/mL, respectively), and were not statistically different from each other (p = 0.48). This reactivity was significantly lower (p < 0.05) for all *Bc* strains and isolates evaluated (e.g. the dairy-isolate strain *Bc* ATCC 10987 had an EC_50_ from 141.89 μg/mL and the *Bc* ATCC 14579 type strain of 223.45 μg/mL). However, all infection-associated *Bc* strains displayed distinctly higher reactivity. For example, the human infection-associated strains *Bc* G9241 and *Bc* 03BB87, Structural Group 2, had approximately 10- to 30-fold lower EC_50_ values and the great ape isolates, Structural Group 3, approximately 2- to 3-fold lower EC_50_ values compared to the non-infection-associated *Bc* strains (p < 0.05). The variance in the EC_50_ values between Structural Group 2 (*Bc* G9241 and *Bc* 03BB87, p = 0.13) and between Structural Group 3 (*Bc* CA and *Bc* CI strains, p = 0.32) were not statistically significant. The HF-SCWP from the highly attenuated *Ba* CDC684 was of particular interest. This SCWP comprised the core HexNAc trisaccharide backbone but was devoid of α- and β-Gal substitutions. This HF-SCWP displayed a significantly higher EC_50_ (lower reactivity) compared to HF-SCWP from *Ba* Sterne 34F2 and *Ba* 7707 (p < 0.05), and from the infection-associated *Bc* G9241, *Bc* 03BB87 and *Bc* CA and CI (p < 0.05). The reactivity of the *Ba* CDC684 isolate was not significantly different to that of the non-infection-associated and structurally unrelated HF-SCWP from *Bc* ATCC 14579 and ATCC 10987 (p = 0.18 and 0.21, respectively; **Table 4**). Essentially, the HF-SCWP from *Ba* CDC684, the great ape *Bc* CA and *Bc* CI isolates, and the *Bc* ATCC 14579 and ATCC 10987 environmental isolates may be considered non-reactive with the mAb (**Fig 7**) although the two great ape isolates did show a slightly elevated reactivity compared to the environmental isolates. Molecular models, generated using the Glycam Webtool [39] with the corresponding force field parameters for carbohydrates [40] yielded several minimal-energy conformers allowing us to compare the epitope presentation for representative SCWP segments from a strong-mAb binder (*Ba* Ames/Sterne), an intermediate mAb binder (human-isolate *Bc* G9241), and a non-binder (*Bc* ATCC 10987) (**Fig 8**). Several minimal energy conformers were generated including *gt-gt* and *gg-gg* forms; only the *gt-gt* conformers are shown. The possible role of these conformers, including the relative importance of individual Gal residues in EAII-6G6 recognition is discussed in **Fig 8**and in the Discussion.

Comparisons of HF-SCWP structures (**Fig 1**) indicate that mAb reactivity can distinguish between the polysaccharide backbones of *Ba* and *Bc* based on the galactosylation pattern of the →4-β-ManNAc-(1→4)-β-GlcNAc-(1→6)-α-GlcNAc-1→ backbone. The absence of Gal residues such as in the *Ba* CDC684 SCWP results in a dramatic loss of binding activity with an EC_50_ value of 356 μg/mL compared to a strain with full galactosylation such as *Ba* Sterne 34F2 with EC_50_ 0.56 μg/mL. The addition of one α-Gal to O3 of the ManNAc residue in *Bc* G9241 SCWP resulted in a statistically significant reduction in binding (EC_50_ 13.6 μg/mL). The addition of a second α-Gal, i.e., as the disaccharide substituent [Gal(α1→3)Gal(α1→], to O3 of the ManNAc residue (refer to **Figs 1 and 2,** or **S2 Fig**) in the SCWP of the *Bc* CI and *Bc* CA isolates resulted in further reductions in binding (EC_50_ 49.94 and 64.34 μg/mL, respectively) of the monoclonal antibody when compared to a monosaccharide α-Gal substitution (**Fig 7**, **Table 4**).

## Discussion

Structural investigations of SCWPs from *Ba* Ames, *Ba* Sterne 34F2, *Ba* 7702, *Ba* Pasteur and *Ba* CDC684, and from infection-associated *Bc* G9241 and *Bc* 03BB87 showed that their SCWPs were comprised of a linear aminoglycosyl (Hex*p*NAc) trisaccharide backbone repeating unit that contains the common structural core motif: →4)-β-D-Man*p*NAc-(1→4)-β-D-Glc*p*NAc-(1→6)-α-D-Glc*p*NAc-(1→ [12,27]. With the exception of the highly attenuated *Ba* CDC684, the GlcNAc residues are highly galactosylated with α- and β-Gal [26,27]. The SCWPs from infection-associated *Bc* strains had an additional α-Gal substitution on the ManNAc backbone residue [27]. Since this aminoglycosyl backbone motif is not present in the non-infection-associated strains *Bc* ATCC 14579 and ATCC 10987, we hypothesized [26] that SCWP molecular structural features (e.g., secondary conformational arrangement and optimal presentation of the Gal substituents) could have an important functional role in pathogen development, including such aspects as wall assembly and cell division/cell chain length. Note that although the HF-SCWP are antigenic, they are not toxic factors and are not considered to directly contribute to *Ba*/*Bc* virulence, however, SCWP may have an indirect role as a result of their known ability to anchor S-layer proteins and direct localization of S-layer associated proteins (e.g. BSL O) to cell septa.

Previous studies by Ezzell *et al*. [19] with mAb EAII-6G6-2-3 and *Ba* derived cell wall fractions showed that its reactivity was inhibited by free monosaccharide galactose and lactose (i.e. Galβ1→4Glc), but not by GlcNAc. This observation suggested to us that the epitope for this antibody may be localized in the SCWP, with particular emphasis on the presentation and arrangement of Gal residues carried on the HexNAc backbone. Modifications in SCWP structures of the *Bc* group strains may therefore be correlated to EAII-6G6-2-3 binding. The recently isolated and phylogenetically well-defined *Bc* CA *and Bc* CI causing anthrax-like symptoms in great apes [15,16] were evaluated to further increase the scope of SCWP structural analysis in infection-associated *Bc*.

The results presented here reveal that the SCWPs isolated from great ape infection- associated *Bc* CA and *Bc* CI isolates had SCWP primary structures almost identical to the SCWP from human infection-associated strain *Bc* G9241 [27] except that a percentage of the backbone ManNAc residues also carry a Galα1→3Galα1→3 disaccharide substituent (**Table 3**). Apart from this disaccharide, the SCWP structures from the *Bc* CA and CI strains contain the structural features previously identified in infection-associated *Ba* and *Bc* strains, i.e. the conserved aminoglycosyl trisaccharide backbone that is substituted with strain-specific galactosylation modifications at conserved locations.

The structural modifications discovered in the SCWPs from the great ape infection- associated *Bc* CA and CI strains compared to the SCWP from *Ba* raised the question whether these modifications affect SCWP immuno-reactivity compared to the those of SCWPs from other infection-associated and non-infection-associated *Bc* group strains [14]. The data from this study indicate that the pAb antiserum did not have sufficient specificity to distinguish between the HF-SCWPs from the *Ba* Ames and human infection-associated isolates *Bc* G9241 and *Bc* 03BB87. The HF-SCWPs from both *Bc* CA and *Bc* CI had similar reactivity, with approximately a 35% reduction in binding of the pAb relative to HF-SCWPs from the *Ba* Ames and human infection- associated isolates *Bc* G9241 and *Bc* 03BB87 (**Fig 6**and **Table 4**). There was a 50% reduction in pAb reactivity to the non-infection-associated strain *Bc* ATCC 10987, and an approximately 80% reduction in reactivity to HF-SCWP from the non-infection-associated strain *Bc* ATCC 14579 and the nongalactosylated aminoglycosyl repeating motif from *Ba* CDC684.

These data suggest that the conserved aminoglycosyl repeating unit backbone, common only to the *Ba* strains and the pathogenic *Bc* isolates, is a predominant antigenic determinant for the pAb, and that their strain specific, α- and β-Gal substitutions at GlcNAc and at ManNAc, confer additional, sometimes unpredictable, polyclonal response. This is emphasized by the fact that a significant response, well above base line, was observed between the polycolonal sera and the HF-SCWP from *Ba* CDC684 (which lacks all Gal substitutions and consists only of the conserved *Ba* aminoglycosyl backbone (**Fig 1**). The polyclonal serum was also somewhat reactive with HF-SCWPs having a structurally different HexNAc backbone, such as those from the nonpathogenic strains *Bc* ATCC 10987 and the type strain *Bc* 14579. These non-infection- associated environmental isolates have HexNAc backbone structures that differ from those shared by the pathogenic *Ba* and *Bc* isolates (**Fig 1**), specifically, [→4)-β-D-Man*p*NAc-(1→4)-β-D-Glc*p*NAc-(1→6)-α-D-Gal*p*NAc-(1→], and both nonpathogenic isolates displayed reduced pAb immunoreactivity. Thus the pAb was able to distinguish between the HF-SCWPs from non-infective isolates and the clinical isolates, although the distinction was not impressive and the pAb response appears to be rather unpredictable, using any straightforward molecular model. It is not clear, for example, why the HF-SCWP from the *Bc* ATCC 10987 and the HF-SCWP from *Ba* CDC684, having different backbone structures, showed very similar immunoreactivity with this polyclonal antiserum. The similar immunoreactivity may be a consequence of both SCWPs containing a conserved →4)-β-D-Man*p*NAc-(1→4)-α-D-Glc*p*NAc-(1→ structural element. Likely, it typifies the broad, non-specific nature of polyclonal sera compared to a mAb, and further investigation is required to define the exact epitope(s) involved in pAb binding.

In contrast to the polyclonal reactivity, the mAb EAII-6G6 had significantly greater ability to discriminate between subtleties in SCWP structure (**Fig 7**). There was approximately a 100-fold reduction in binding of the mAb to HF-SCWPs from both *Bc* CA and *Bc* CI, relative to HF-SCWPs from the *Ba* Sterne 34F2 or *Ba* 7702, and a 20-fold reduction in reactivity compared to human infection-associated isolates *Bc* G9241 and *Bc* 03BB87. Small differences in mAb reactivity between isolates *Bc* G9241 and *Bc* 03BB87 were also detected, however, these differences could simply result from experimental variability, for instance, differences in purity of the two preparations. All of the 2-D NMR signals arising from each of these two *Bc* human infection-associated SCWPs were essentially indistinguishable [27], suggesting they are of identical structure. However low levels of insoluble contaminants (e.g. peptidoglycan fragments), and low levels of a contaminating glycogen polymer vary slightly among different preparations, and their presence could likely account for the slight displacement of these two binding curves (**Fig 7**). Reactivity of the mAb to the non-galactosylated aminoglycosyl repeating motif from *Ba* CDC684 was reduced more than 600-fold compared to *Ba* Sterne 34F2 or *Ba* 7702. Reactivity to the differing HF-SCWP structures from *Bc* ATCC 10987 and *Bc* ATCC 14579 was reduced by 250- and 400-fold, respectively. Thus, the observed reactivity of EAII-6G6 with the examined HF-SCWPs (**Fig 7**) suggests that four main structural groups can be distinguished with this mAb: 1) non-reactive (*Ba* CDC684 and the non-pathogenic *Bc* ATCC 10987 and *Bc* ATCC 14579, 2) low reactive (both great ape isolates (*Bc* CA and *Bc* CI), 3) intermediate reactivity (human infection-associated *Bc* G9241 and *Bc* 03BB87), and 4) highly reactive (*Ba* Ames, Pasteur, Sterne, and related examined *Ba* strains). The structural basis of this reactivity is suggested to reside in the presentation of multiple Gal residues in the proper 3-dimensional conformation. This 3-dimensional arrangement (secondary structure) is optimal only in the *Ba* derived strains, (with the exception of *Ba* CDC684 which lacks galactose, **Fig 1**). The introduction of an additional Gal residue, at O-3 of the backbone ManNAc residue (i.e., for *Bc* G9241 and *Bc* 03BB87), disrupts this secondary structure of the polysaccharide sufficiently, such that EAII-6G6 binding is reduced. The attachment of *further* additional Gal, (i.e., in the form of Galα1→3Galα1→ disaccharide to ManNAc in both great ape isolates) causes further deviation from the optimal secondary structure necessary for mAb recognition, yielding the weak binding observed with this structural group. One possibility is that approach of the mAb to the multiple Gal epitope presented by the SCWP is simply hindered by the presence of the new disaccharide (and monosaccharide) Gal substituents, at ManNAc in these strains, as visualized by molecular models (**Fig 8**). Alternatively, the presence of these new substituents could also result in a change in the overall secondary conformation presented by the SCWP, compared to that of *Ba* Ames, Pasteur, Sterne, and related *Ba* strains. A combination of these factors may also account for the observed binding differences. It is interesting to note that HF-SCWPs derived from the nonpathogenic strains (e.g., *Bc* ATCC 10987 and *Bc* ATCC 14579), which do not contain the conserved HexNAc trisaccharide backbone of the infection-associated strains, elicit binding essentially at baseline levels, similar to BSA. The *Ba* CDC684 HF-SCWP, which *does* contain the pathogenicity-associated HexNAc backbone but lacks all Gal substituents, also shows this same lack of reactivity with the EAII-6G6 mAb. Thus, both the pathogenicity-associated HexNAc trisaccharide backbone, together with the optimal pattern of α/β- attached Gal residues (found only in *Ba* strains), is required to yield the maximum binding of EAII-6G6. Molecular models generated using the Glycam web tool [39] with standard Glycam06 force field parameters [40] yielded several low energy conformers for SCWPs from three representative strains, *Ba* (Ames), *Bc* G9241, and *Bc* ATCC 10987 (strong, intermediate, and non-binding) as compared in **Fig 8**. These models reveal how additional Gal substituents located at ManNAc could perturb mAb binding and accessibility to the correct Gal epitope. For instance, **Fig 8**shows identical views of a minimal energy *gt-gt* conformer for an eleven (or twelve) residue segment for both the *Ba* Sterne 34F2 and the *Bc* G9241 HF-SCWPs. The backbone segments depicted are of the sequence: **C-A-B-C-A**, with ManNAc (residue **B**), depicted in the middle of the segment since this ManNAc residue is the focal point of the structural difference. Of interest is the close proximity of the α-Gal (**E**) and β-Gal (**F**) substituents at the 6-linked GlcNAc (residue **A**) to each other, as well as their close proximity to the α-Gal monosaccharide substituent (residue **G**) at ManNAc in the *Bc* G9241 segment. We propose that accessibility of these two Gal residues (**E** and **F**) to the mAb could be reduced by the presence of a monosaccharide Gal substituent at ManNAc (as in the human isolate *Bc* G9241), and further reduced by the presence of a Galα1→3Gal disaccharide at ManNAc, resulting in the weaker binding for the great ape isolates (**Fig 7**). The particular low energy conformer depicted in **Fig 8**also suggests the possibility that exposed portions of the two closely located Gal residues (**E** and **F**) might contribute, perhaps equally, to the EAII-6G6 epitope. Note also that the SCWP from *Bc* ATCC 10987 does indeed carry a β-Gal substituent, yet is essentially non-reactive with the mAb EAII-6G6 (**Fig 7, Table 4**). This observation is consistent with the hypothesis that the *Ba*-conserved “pathogenicity motif” backbone is essential in presenting the Gal substituents to the mAb, by providing the proper conformational scaffolding. Although not proven here, we suggest the possibility that the combined arrangement of both Gal residues **E** and **F** may contribute to the EAII-6G6 epitope. The HexNAc backbone itself probably does not interact directly with the mAb, (note the non-binding observed with the *Ba* CDC684 SCWP, **Fig 7**), however, this pathogenicity-specific backbone is necessary to provide the proper scaffolding for presentation of the multiple Gal epitopes which do interact with the mAb. The models provide a visual assist to our central hypothesis, that the correct number of Gal residues, carried on the proper HexNAc backbone at specific locations, are required for optimal mAb EAII-6G6 recognition. Removal or addition of Gals or any change in the Gal attachment pattern, from that displayed in *Ba* strains, results in variable degrees of attenuation of mAb binding.

The aminogycosyl backbone is also likely to be of importance for the SCWP immunoreactivity with the mAb, as shown with the nearly abrogated reactivity of SCWP samples from the non-infection-associated *Bc* isolates. In contrast to the conserved core repeating trisaccharide backbone of the infection-associated *Ba* and *Bc* strains, the HF-SCWPs of *Bc* ATCC 10987 and the *Bc* type strain ATCC 14579 possessed entirely different backbone repeating structures [13, 42]. Presumably due to the differences of the aminoglycosyl backbones of these strains, they presented significantly higher EC_50_ values when compared to the *Ba* Sterne 34F2 and *Ba* 7702 strains.

The EAII-6G6-2-3 antibody has been used in diagnostic tests and to probe for the distribution of the EAII-6G6-2-3 epitope in SCWPs of *Bc* group strains. De *et al*. analyzed 230 *Ba* isolates with a two component direct fluorescent antibody (DFA) assay using fluorescein labeled EAII-6G6-2-3 against the cell wall polysaccharide antigen and a monoclonal antibody against the capsule antigen [18]. Of the 230 *Ba* isolates tested, 228 were positive for reactivity with EAII-6G6. The assay specificity for EAII-6G6-2-3 was only 78.6%, and almost 100% of the false-positive isolates were *Bc* or *Bacillus thuringiensis* strains. Ten of 23 (43%) *B*. *cereus* strains were reported positive for the cell wall antigen. Further DFA analysis of *B*. *cereus* strains G9241 and 03BB87 were also positive for cell wall polysaccharide antigen [22, 24], whereas that of *Bc* ATCC 14579 was negative [18].

We suggest that the mAb antibody is recognizing a specific epitope in the cell wall polysaccharide antigen, which is impacted by number and position of galactose modifications. While we have shown that removal of all Gal residues (*Ba* CDC684) causes loss of binding and the addition of Gal to ManNAc reduces binding (*Bc* G9241/03BB87, and *Bc* CA and CI), we do not yet know the specific contributions of each of the three Gal residues in the *Ba* structure that provides optimal binding. Our minimal energy models (**Fig 8**) suggest a preliminary possibility that Gal residues **E** and **F** may together contribute to the EAII-6G6 epitope. A detailed analysis of the minimal *Ba* SCWP epitope and Gal positions required for EAII-6G6-2-3 binding is being investigated by preparing synthetic structural analogs. Further, while we know that the terminal repeat unit is pyruvylated, O-acetylated, and de-*N*-acetylated [26], we do not know the extent of their contribution, if any, to the mAb-epitope. However, all of our data suggest that these non-carbohydrate substituents are not key elements of the EAII-6G6 epitope. For instance, HF-SCWPs from all of the examined strains, (all pathogenic *Ba* strains, the *Ba* CDC684 non galactosylated strain, the human isolates *Bc* G9241 and *Bc* 03BB87, the great ape isolates *Bc* CA and CI, and both ATCC environmental strains), carry a pyruvate residue at identical location on the terminal repeating unit as reported here (**S3, S5**and **S7 Figs**) and discussed in (26), however, only the galactosylated strains *containing the pathogenicity-associated HexNAc backbone* demonstrate significant EAII-6G6 binding.

It has been established that cell wall polysaccharides can be virulence factors in both Gram-positive and Gram-negative pathogens [43–45] and are used as diagnostic targets [46,47] or as carbohydrate-based vaccine antigens [48,49]. For instance, the galactomannan cell wall polysaccharide of *Aspergillus fumigatus*, an opportunistic fungal pathogen, is an antigen that is the basis for a diagnostic test against the tetra-β-(1→5) galactofuran chain [50]. As such, cell wall polysaccharides amongst the closely related *B*. *cereus* group may be useful in development of rapid diagnostic tests for inhalation anthrax.

Monoclonal antibody EAII-6G6-2-3 has been used and previously published as a differential diagnostic reagent for *B*. *anthracis* [18], and it is still used at CDC for immunohistochemistry tests in tissue. We demonstrated that the ability of mAb EAII-6G6-2-3 to distinguish between disease causing strains of the *Bc* group is based on the number and position of galactosyl modifications around a secondary cell wall polysaccharide backbone. So far, in *Bacillus* species capable of causing infection this polysaccharide was comprised of →4)-β-D-Man*p*NAc-(1→4)-β-D-Glc*p*NAc-(1→6)-α-D-Glc*p*NAc-(1→ and we propose that this aminoglycosyl core constitutes a pathogenicity-related motif associated with *Bacillus* infectivity. In addition, a putative association between pathogenicity and the galactosyl modifications is emerging. Based on the reported *in vivo* activities of the various strains evaluated here, the presence and relative positions of the modifications may be associated with relative infectious potency. For example, all the strains containing the →4)-β-D-Man*p*NAc-(1→4)-β-D-Glc*p*NAc-(1 →6)-α-D-Glc*p*NAc-(1→ core motif are associated with human disease or capable of causing fatal infection in animal models. However, the *Ba* CDC684 strain lacking galactosyl residues is highly attenuated in a guinea pig model of infection whereas the galactosylated *Ba* Sterne 34F2 and *Ba* 7702, although lacking the poly-D-glutamic acid capsule virulence factor, retain a significant level of virulence in mouse and rabbit models of anthrax [51]. The accumulating data are increasingly indicative of the aminoglycosyl core motif as a marker of pathogenicity and it is tempting to speculate that the core motif in its glycosylated form may be a putative pathogen associated molecular pattern that enhances the capability of the organism to cause infection.

## Supporting information

S1 Fig Partial ^1^H-^13^C HSQC spectra comparing the anomeric regions of representative HF-SCWP from human infection-associated *Bacillus* strains. **(A)** *Bc* G9241; **(B)** *B*. *anthracis* Sterne 34F2. For complete details of this structural comparison see [27] Forsberg, et al., *Glycobiology* (2011) 21:934–948. The anomeric signals circled in red are unique to the human infection-associated *Bc* strains, and *absent* from *Ba* Sterne, Ames, and Pasteur strains. Their presence reflects an alteration in magnetic environment due to 3-O-substitution of 50% of the ManNAc residues (**B′**) with αGalρ residue (**G**) in the *Bc* G9241 SCWP; the α/β-GlcNAc signals arise from the reducing-end residue of the HF-released SCWPs [27].
(TIF)

S2 Fig Structural relationship and comparison of the ^1^H-NMR anomeric regions for HF-SCWPs from *B*. *anthracis* Sterne, the representative human infection-associated *B*. *cereus* strain G9241, and the great ape isolates *B*. *cereus* CA and *Bc* CI. New anomeric signals (from residues **J** and **K**) are indicated. Signals having a unique downfield shift, diagnostic for 3-substituted ManNAc (**B′3**, **B′′3**) are also labeled and are present only in the *Bc* infection-associated strains (refer to **Table 2**).
(TIF)

S3 Fig **Comparison of** ^**1**^**H-**^**1**^**H-TOCSY spectra for HF-SCWPs from (A) *B*. *cereus* CA (Cameroon), and (B) human infection-associated *B*. *cereus* G9241.** Consistent with **S2**, **S4**, **S7**and **S8 Figs** the TOCSY spectra from these two strains are virtually identical and superimposable, except for the presence of the additional residues **J** and **K** in *Bc* CA SCWP**.** In *panel A*, the (red box) shows the TOCSY region expanded in **S6A Fig**. In *panel B*, the human isolate *Bc* G9241 lacks the **J** and **K** spin systems. All other TOCSY connectivities detected in the great ape *Bc* CA and *Bc* CI isolates are virtually identical to those from the human infection-associated HF-SCWPs; these signals arise from the conserved residues that are shared with, and previously identified [27] for the human infection-associated strains (*Bc* G9241/*Bc* 03BB87/*Bc* 03BB102), as represented in *panel B*. The chemical shifts of these conserved residues are remarkably similar in these HF-SCWPs. For example, all examined HF-SCWPs show connectivities arising from the α- and β- reducing end GlcNAc residue on each polysaccharide (labeled α/β). These weak spin systems are listed in the **Table 2***footnote*: *“Additional Signals”*. Positional assignments from COSY analysis (not shown) have been previously published for this reducing end residue from *Ba* and *Bc* strains [26,27]. Their resonances are virtually superimposable and show very little variation in each polysaccharide examined, including the great ape isolates described here. Also refer to the ^1^H-^13^C-HSQC in **S7 Fig**, which shows the H2/C2 correlation for the reducing end residue. *Other substoichiometric signals*—Residue **C′** is a 3-*O*-acetylated variant of **C**, which also occurs at a specific location in each HF-SCWP molecule from all examined strains [26]. Shown is the H3 proton of this residue, a doublet of doublets; also note its presence as a “reporter signal” in **S5 Fig**, showing the ^1^H-^13^C HSQC (H3/C3 δ_H_/δ_C_ 5.09/74.2). Residue **C′** was first identified in the *B*. *anthracis* strain CDC684 HF-SCWP [26] and was subsequently detected in *Ba* Sterne 34F2, *Bc* G9241 [26], and in the present work in both great ape-derived *Bc* strains. Of further interest, in all examined HF-SCWPs the attached Gal substituents **D**, **E**, and **F** all display signal heterogeneity, which appears to arise from nonstoichiometric Gal attachment in some of the repeating units. The weak system at δ_H_ 5.39 (*) arises from the anomeric proton of 4-linked α-glucose residues, due to variable amounts of a contaminating glucan produced by the bacteria. The majority of this glucan was removed prior to NMR analysis by Superose-12 chromatography (**S12 Fig**) as previously described for *Ba* and *Bc* SCWPs [26, 27].
(TIF)

S4 Fig Comparison of ^1^H-^1^H-TOCSY partial spectra for HF-SCWPs from the great ape isolates. (**A)** *B*. *cereus* CA (Cameroon), and (**B)** *B*. *cereus* CI (Côte d’Ivoire). These TOCSY spectra are virtually superimposable, reflecting the structural similarity of the SCWPs from these two great ape isolates. This is consistent with the ^1^H anomeric NMR spectra (**S2 Fig**.) which indicated differences only in signal area for these two strains, with identical chemical shifts.
(TIF)

S5 Fig Comparison of the ^1^H-^13^C-HSQC anomeric regions for native and borodeuteride-reduced *B*. *cereus* Cameroon HF-SCWPs. ***A***, native *Bc* CA strain HF-SCWP, and ***B***, the same sample reduced with borodeuteride to remove the anomeric signal from the reducing end (converting this residue to alditol) allowing elucidation of the **K** residue system. ***Arrows*** show the location of both α- and β- reducing end (GlcNAc) anomeric signals prior to reduction; note their absence in the red-HF-SCWP (*panel* ***B***). These spectra demonstrate that reduction did not appear to affect any other residues, only the free reducing end. The locations of new residues **J** and **K** (anomeric signals) and all other anomeric signals from the repeating units were unchanged. Note also the presence the of δ_H_ downfield-shifted O-acetyl “reporter” signal, which arises from the H3 proton on a 3-O-acetylated β-GlcNAc residue (**C′**); this O-acetylated GlcNAc residue occurs at a specific, non-repeated location in all *Ba* and pathogenic *Bc* derived HF-SCWPs examined to date [26]. The scalar connectivities for this residue are visible in **S3 Fig**. A detailed description of the location and identity of this and other substoichiometric modifications is presented in Forsberg *et al*., [26].
(TIF)

S6 Fig **Comparison of** ^**1**^**H-**^**1**^**H TOCSY spectra for the *Bc* CA HF-SCWP showing the region containing residues J, K, and G, before (*A*), and after (*B*) reduction of the reducing end of the SCWP with borodeuteride.** In *panel A*, **α** = α-reducing end GlcNAc anomeric proton. Certain scalar correlations arising from this α-reducing end anomeric proton overlapped with some of the **K** residue protons; removal of these α-reducing end signals by borodeuteride reduction allowed definitive assignment of the **K** ring system (*panel B*). The β-GlcNAc reducing end anomeric is found at δ_H_ 4.74 (refer to **S3**, **S5A, and S8 Figs** and the **Table 2**footnote: “Additional Signals”).
(TIF)

S7 Fig **Partial** ^**1**^**H-**^**13**^**C HSQC spectra comparing HF-SCWPs from *Bc* strains: (A) human isolate *Bc* G9241; (B) great ape isolate *Bc* CA (Cameroon).** Proton multiplicity is indicated by peak polarity, i.e., orange/red gradient signals (**positive polarity**) arise from methyl or methine ring protons, and blue/pink gradient signals **(negative polarity**) arise from methylene (-CH_2_-) protons. Distinct signals arising from position H6/C6 of residue **J** (“**J6**”) were observed in *Bc* CA (also *Bc* CI, not shown), which were absent in the human Infection-associated *Bc* G9241 and *Ba* strains (previously published [26,27]). In addition, a ^1^H-^13^C H2BC analysis provided 2-bond couplings which assisted in residue **J** assignments (**S10 Fig**). Pyruvate "reporter signals" and the location of pyruvate in HF-SCWPs from representative *Ba* and *Bc* strains is discussed in detail in Forsberg *et al*., 2012 [26]. Briefly, pyruvate ketal is linked to positions 4 and 6 of the terminal, non-reducing end ManNAc residue, designated **B′**. This substitution precludes further chain polymerization. At **B′** positions 4, 5, and 6, unique δ_H_/δ_C_ result from this pyruvate substitution (yielding unique “reporter signals”). A 3-bond ^1^H-^13^C HMBC coupling was also observed between the pyruvate C2 carbon, and a favorably oriented H6 proton of **B′** for all examined HF-SCWP [26]. These and other reporter signals, arising from substoichiometric modifications (e.g., O-acetylation), occur at virtually identical resonance in HF-SCWPs from all examined strains of *Ba* and *Bc*.
(TIF)

S8 Fig **Comparison of the** ^**1**^**H-**^**13**^**C HSQC anomeric regions of *Bc* HF-SCWPs from: (A) *Bc* G9241; (B) *Bc* CA (Cameroon).** The spectra are virtually superimposable, with the exception of "new" anomeric signals from residues **J** and **K** in the *Bc* CA sample. Additional heterogeneity of residue **B** (residue **B′′**) is also observed in *Bc* CA (and *Bc* CI) SCWPs. The α- and β- labels designate the anomeric signals arising from the reducing-end GlcNAc residue of the respective HF-SCWPs.
(TIF)

S9 Fig Partial ^1^H-^13^C HMBC spectrum showing the trans-glycosidic connectivities between J1→B′′3 and G1→B′3 for the borodeuteride reduced-HF-SCWP from great ape isolate *Bc* CA. These 3-bond connectivities prove the linkage of residue **J** to the 3-position of residue **B′′**, and of residue **G** to 3-position of **B′**.
(TIF)

S10 Fig ^1^H-^13^C HSQC and ^1^H-^13^C H2BC spectral regions demonstrating features of the new J and K residue spin systems, residues unique to the *Bc* great ape strain isolates. *Panel A*, HSQC, and *panel B*, H2BC of the *Bc* CA red-HF-SCWPs.
(TIF)

S11 Fig Linkage (methylation) analysis of HF-SCWPs from *Bc* G9241 and *Bc* CA (Cameroon). **(A)** Total ion chromatogram (TIC) of the neutral sugar PMAA derivatives from *Bc* G9241 SCWP; **(B)** TIC of neutral sugar PMAAs from *Bc* CA HF-SCWP; **(C)** electron impact mass spectrum of the 3-linked Gal PMAA derivative from the *Bc* CA sample. The GC-MS analysis of the neutral sugar PMAA derivatives demonstrates the presence of 3-linked Gal in the "great ape" strain HF-SCWP, but not in *Bc* G9241. Only the great ape derived SCWPs (from *Bc* CA and *Bc* CI) yield this derivative, which arises from the **K**1**→J**3 disaccharide substituent unique to these strains. The results of this chemical analysis (1.86 residues/PS for *Bc* CA) are in close agreement with NMR signal integration (refer to **Table 3**). The 4-linked-Glc derivative arises from traces of a contaminating α-glucan, most of which is removed by SEC (see **S12 Fig**).
(TIF)

S12 Fig Purification and molecular size estimation of the HF-SCWP isolated from different *Bacillus* strains. SCWP were released from cell walls by treatment with hydrofluoric acid, dialyzed, then chromatographed on a Superose-12 FPLC size exclusion column with comparison to commercially available α1**→**6 linked dextrans. **A**, great ape isolate *B*. *cereus* CA (Cameroon) (Kav 0.410). **B**, *B*. *anthracis* Sterne 34F2 (Kav 0.433). **C**, type strain *B*. *cereus* ATCC14579 (Kav 0.337; calculated mass 20,020 Da). The HF-SCWP from the great ape isolates *Bc* CA/CI and *Bc* strains all elute at approximately the same location, between the 25 kDa and 10.5 kDa dextran standards, having calculated mass of approx. 12,000 Da for *Bc* CA (Kav 0.410) and for *Bc* G9241 (Kav 0.411, not shown). The elution profiles of human isolates *Bc* G9241 and *Bc* 03BB87 HF-SCWPs are all essentially identical to that of *Bc* CA ([27], see Forsberg et al., 2011, Supplement Data for human *Bc* isolate analysis). Interestingly, the structurally unrelated *B*. *cereus* type strain ATCC14579 HF-SCWP elutes slightly after the 25 kDa dextran standard suggesting a mass around 20 kDa. The *Bacillus* SCWP isolates, in particular those from *Ba* and pathogenic *Bc*, contain variable amounts of a high molecular weight glucan which elutes at the void volume (**Vo**) (panels **A** and **B**). Glycosyl composition (GC-MS) and NMR analysis (COSY, TOCSY, HSQC) indicated that this void volume polysaccharide is composed exclusively of glucose in predominately α1**→**4 linkage. This glucan may correspond to a glycogen polysaccharide previously reported in certain *B*. *anthacis* and related pathogenic *Bacillus* strains. Proteins (i.e., aromatic compounds, Abs 280 nm) also elute at this void region. Column total inclusion volume = (**Vi**). In addition to the 35–40 kDa dextran profile, the elution positions of the 25 kDa and 10.5 kDa dextrans are indicated by *arrows* (**1** = 35–40 kDa, Kav 0.239; **2** = 25 kDa, Kav 0.299; **3** = 10.5 kDa, Kav 0.433).
(TIF)

## Abbreviations

ATCC: American Type Culture Collection
*Bc* CA: *B*. *cereus strain Cameroon (CA)*
*Bc* CI: *B*. *cereus strain Côte d*’Ivoire (CI)
BcG9241: *B*. *cereus strain G9241*
Ba684: *B*. *anthracis CDC 684*
BSL: *Bacillus S-layer associated protein*
COSY: ^1^H-^1^H correlation spectroscopy
DSS: 2,2-dimethyl-2-silapentane-5-sulfonate, sodium salt
gt-gt: *gauche-trans*
GC-MS: gas-liquid chromatography-mass spectrometry
HF: hydrofluoric acid
H2BC: heteronuclear 2-bond correlation spectroscopy
HF-SCWP: SCWP released from peptidoglycan by HF treatment
HMBC: heteronuclear multiple bond coherence spectroscopy
HSQC: heteronuclear single quantum coherence spectroscopy
NOESY: nuclear Overhauser effect spectroscopy
PG: peptidoglycan
PMAA: partially methylated alditol acetate
Pyr: pyruvate
red-HF-SCWP: HF-released SCWP treated with borodeuteride to reduce the reducing end
SCWP: secondary cell wall polysaccharide
SEC: size exclusion chromatography
SLH: surface layer homology
TMS: trimethylsilyl
TOCSY: total correlation spectroscopy
