# Supplementary figures and images for: Structural and immunochemical relatedness suggests a conserved pathogenicity motif for secondary cell wall polysaccharides in Bacillus anthracis and infection-associated Bacillus cereus
Source: PLoS One. 2017 Aug 23;12(8):e0183115. doi: 10.1371/journal.pone.0183115 (PMC5568421; doi:10.1371/journal.pone.0183115)

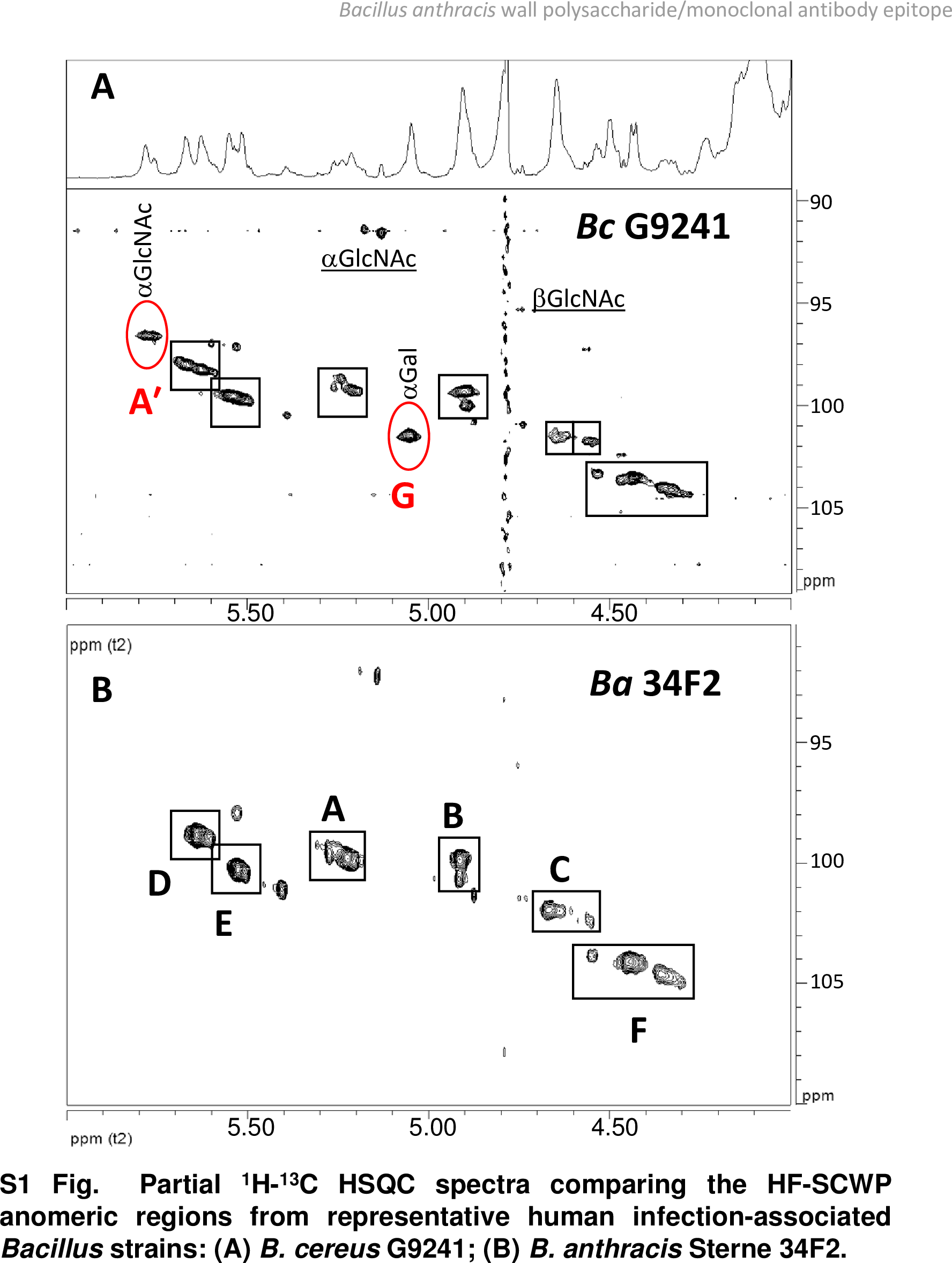

Supplement: S1 Fig — (A) Bc G9241; (B) B. anthracis Sterne 34F2. For complete details of this structural comparison see [27] Forsberg, et al., Glycobiology (2011) 21:934–948. The anomeric signals circled in red are unique to the human infection-associated Bc strains, and absent from Ba Sterne, Ames, and Pasteur strains. Their presence reflects an alteration in magnetic environment due to 3-O-substitution of 50% of the ManNAc residues (B′) with αGalρ residue (G) in the Bc G9241 SCWP; the α/β-GlcNAc signals arise from the reducing-end residue of the HF-released SCWPs [27]. (TIF) [file pone.0183115.s001.tif]

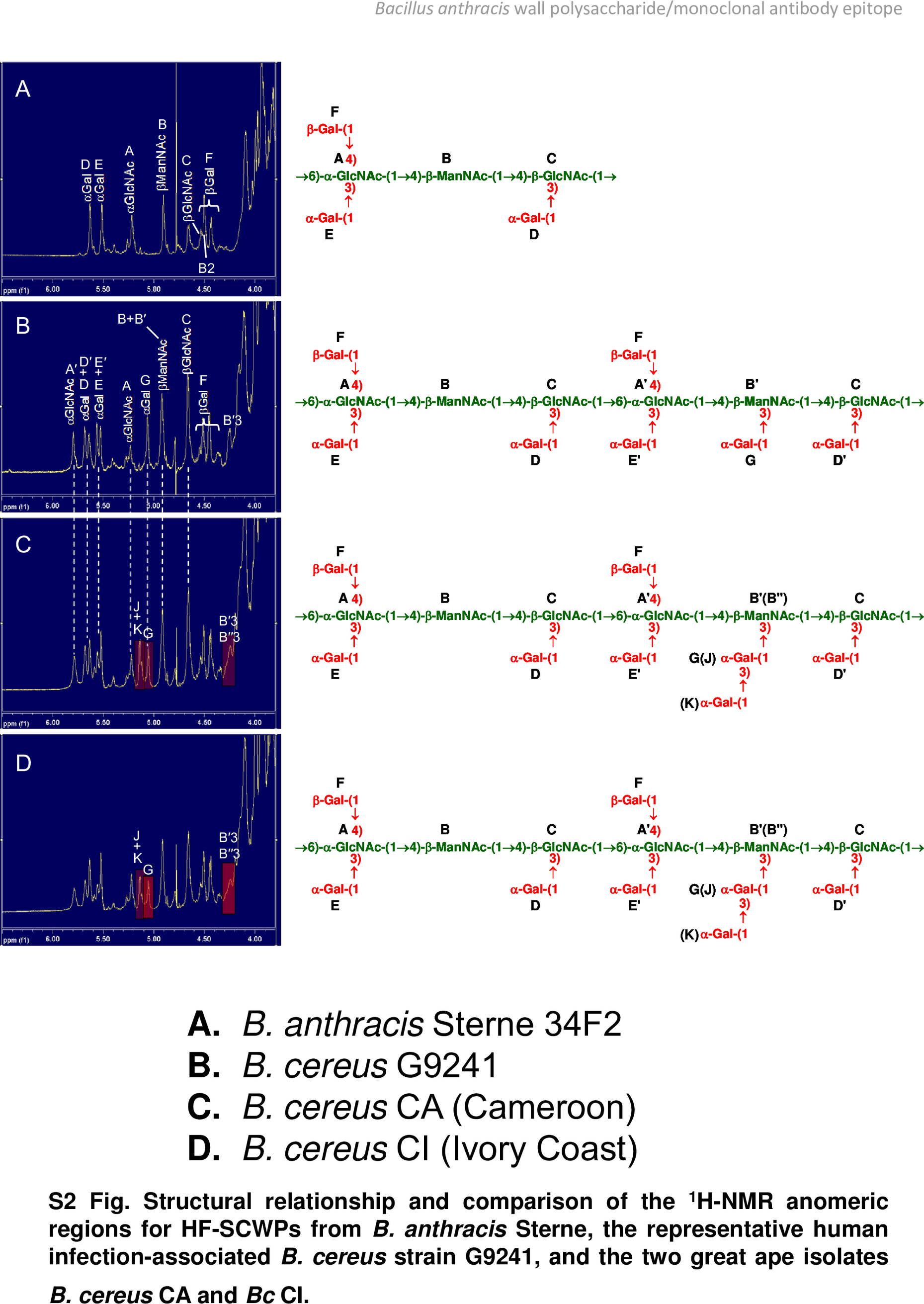

Supplement: S2 Fig — New anomeric signals (from residues J and K) are indicated. Signals having a unique downfield shift, diagnostic for 3-substituted ManNAc (B′3, B′′3) are also labeled and are present only in the Bc infection-associated strains (refer to Table 2). (TIF) [file pone.0183115.s002.tif]

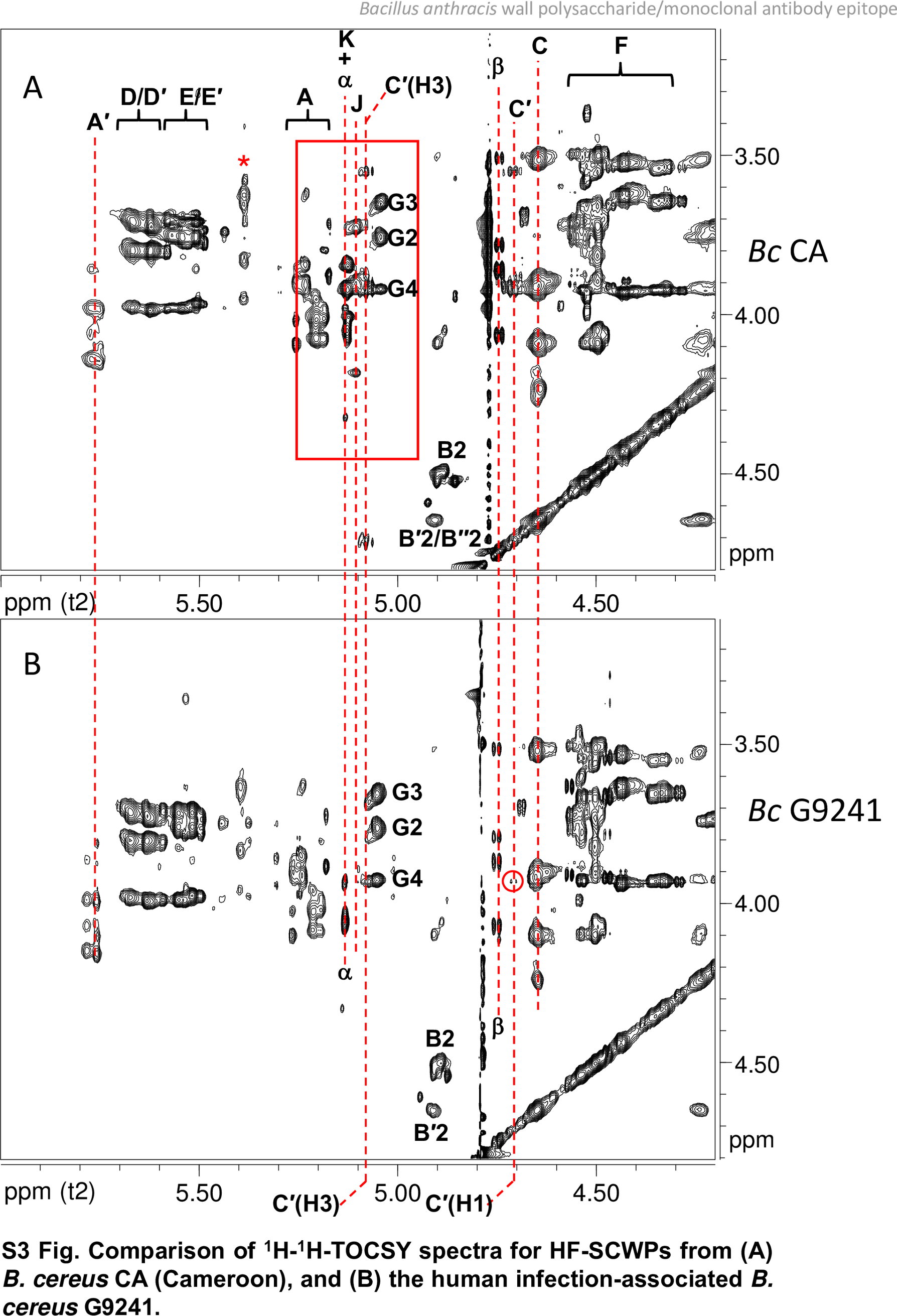

Supplement: S3 Fig — Comparison of 1H-1H-TOCSY spectra for HF-SCWPs from (A) B. cereus CA (Cameroon), and (B) human infection-associated B. cereus G9241. Consistent with S2, S4, S7and S8 Figs the TOCSY spectra from these two strains are virtually identical and superimposable, except for the presence of the additional residues J and K in Bc CA SCWP. In panel A, the (red box) shows the TOCSY region expanded in S6A Fig. In panel B, the human isolate Bc G9241 lacks the J and K spin systems. All other TOCSY connectivities detected in the great ape Bc CA and Bc CI isolates are virtually identical to those from the human infection-associated HF-SCWPs; these signals arise from the conserved residues that are shared with, and previously identified [27] for the human infection-associated strains (Bc G9241/Bc 03BB87/Bc 03BB102), as represented in panel B. The chemical shifts of these conserved residues are remarkably similar in these HF-SCWPs. For example, all examined HF-SCWPs show connectivities arising from the α- and β- reducing end GlcNAc residue on each polysaccharide (labeled α/β). These weak spin systems are listed in the Table 2footnote: “Additional Signals”. Positional assignments from COSY analysis (not shown) have been previously published for this reducing end residue from Ba and Bc strains [26,27]. Their resonances are virtually superimposable and show very little variation in each polysaccharide examined, including the great ape isolates described here. Also refer to the 1H-13C-HSQC in S7 Fig, which shows the H2/C2 correlation for the reducing end residue. Other substoichiometric signals—Residue C′ is a 3-O-acetylated variant of C, which also occurs at a specific location in each HF-SCWP molecule from all examined strains [26]. Shown is the H3 proton of this residue, a doublet of doublets; also note its presence as a “reporter signal” in S5 Fig, showing the 1H-13C HSQC (H3/C3 δH/δC 5.09/74.2). Residue C′ was first identified in the B. anthracis strain CDC684 HF-SCWP [26] and was sub [file pone.0183115.s003.tif]

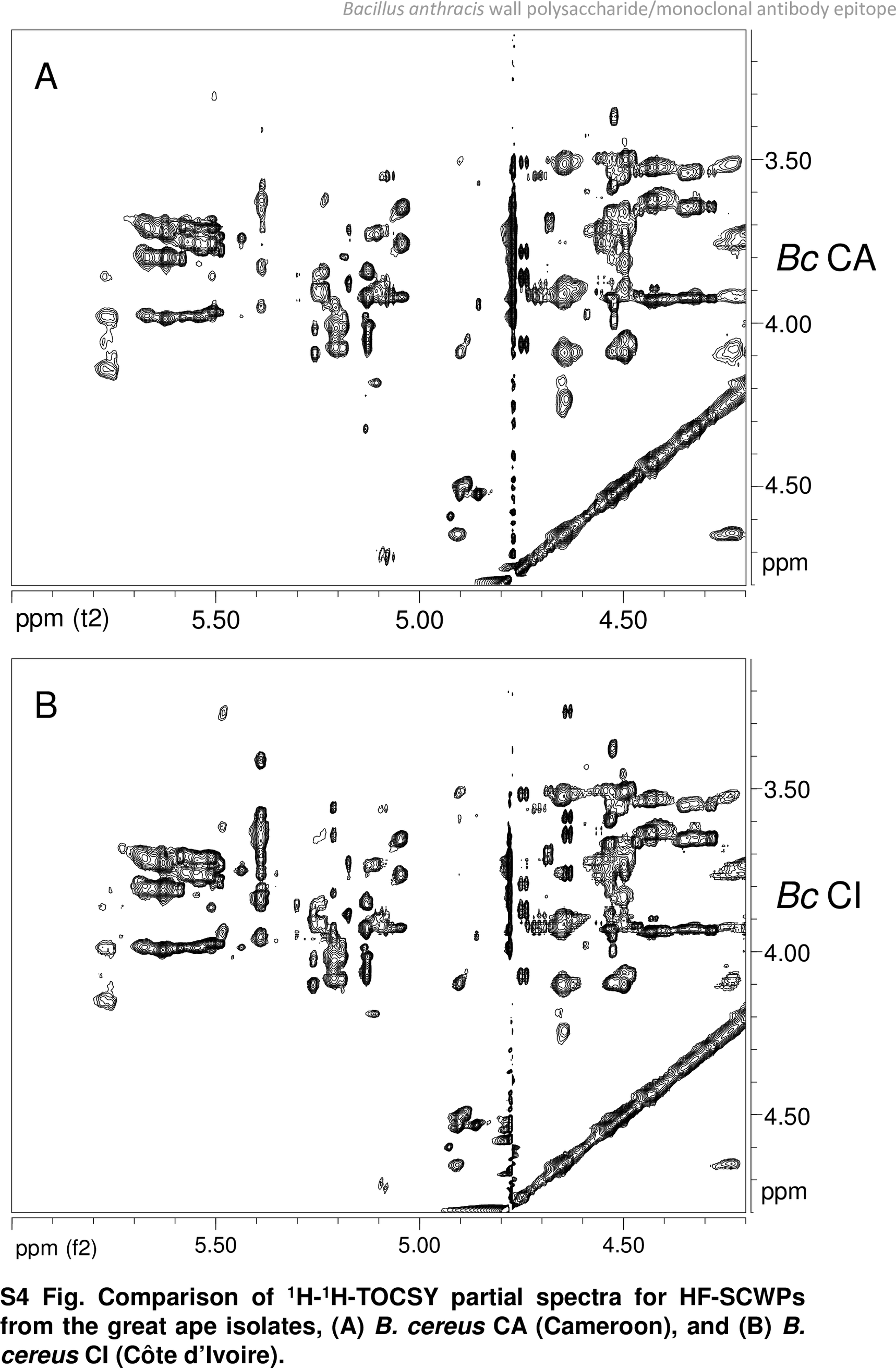

Supplement: S4 Fig — (A) B. cereus CA (Cameroon), and (B) B. cereus CI (Côte d’Ivoire). These TOCSY spectra are virtually superimposable, reflecting the structural similarity of the SCWPs from these two great ape isolates. This is consistent with the 1H anomeric NMR spectra (S2 Fig.) which indicated differences only in signal area for these two strains, with identical chemical shifts. (TIF) [file pone.0183115.s004.tif]

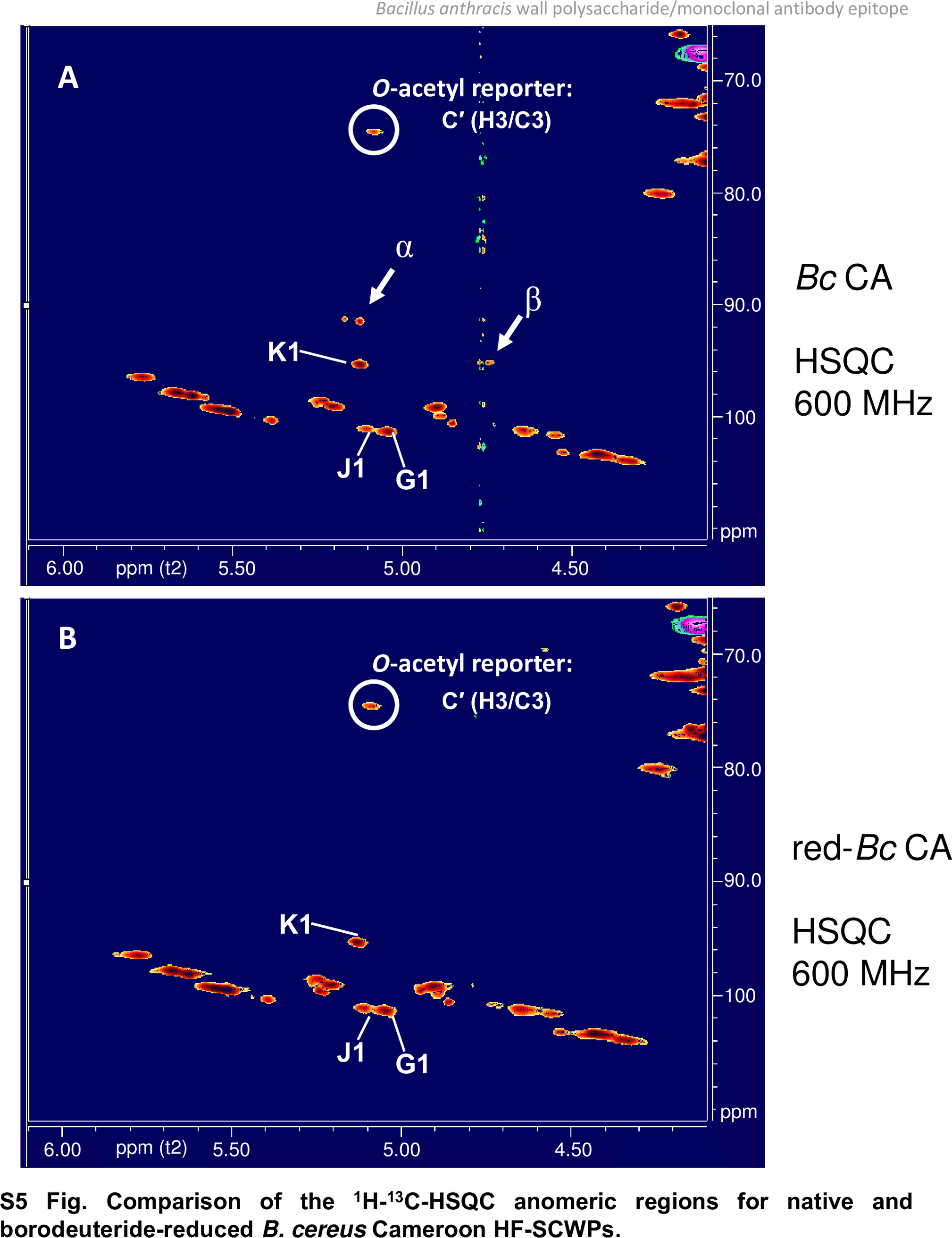

Supplement: S5 Fig — A, native Bc CA strain HF-SCWP, and B, the same sample reduced with borodeuteride to remove the anomeric signal from the reducing end (converting this residue to alditol) allowing elucidation of the K residue system. Arrows show the location of both α- and β- reducing end (GlcNAc) anomeric signals prior to reduction; note their absence in the red-HF-SCWP (panel B). These spectra demonstrate that reduction did not appear to affect any other residues, only the free reducing end. The locations of new residues J and K (anomeric signals) and all other anomeric signals from the repeating units were unchanged. Note also the presence the of δH downfield-shifted O-acetyl “reporter” signal, which arises from the H3 proton on a 3-O-acetylated β-GlcNAc residue (C′); this O-acetylated GlcNAc residue occurs at a specific, non-repeated location in all Ba and pathogenic Bc derived HF-SCWPs examined to date [26]. The scalar connectivities for this residue are visible in S3 Fig. A detailed description of the location and identity of this and other substoichiometric modifications is presented in Forsberg et al., [26]. (TIF) [file pone.0183115.s005.tif]

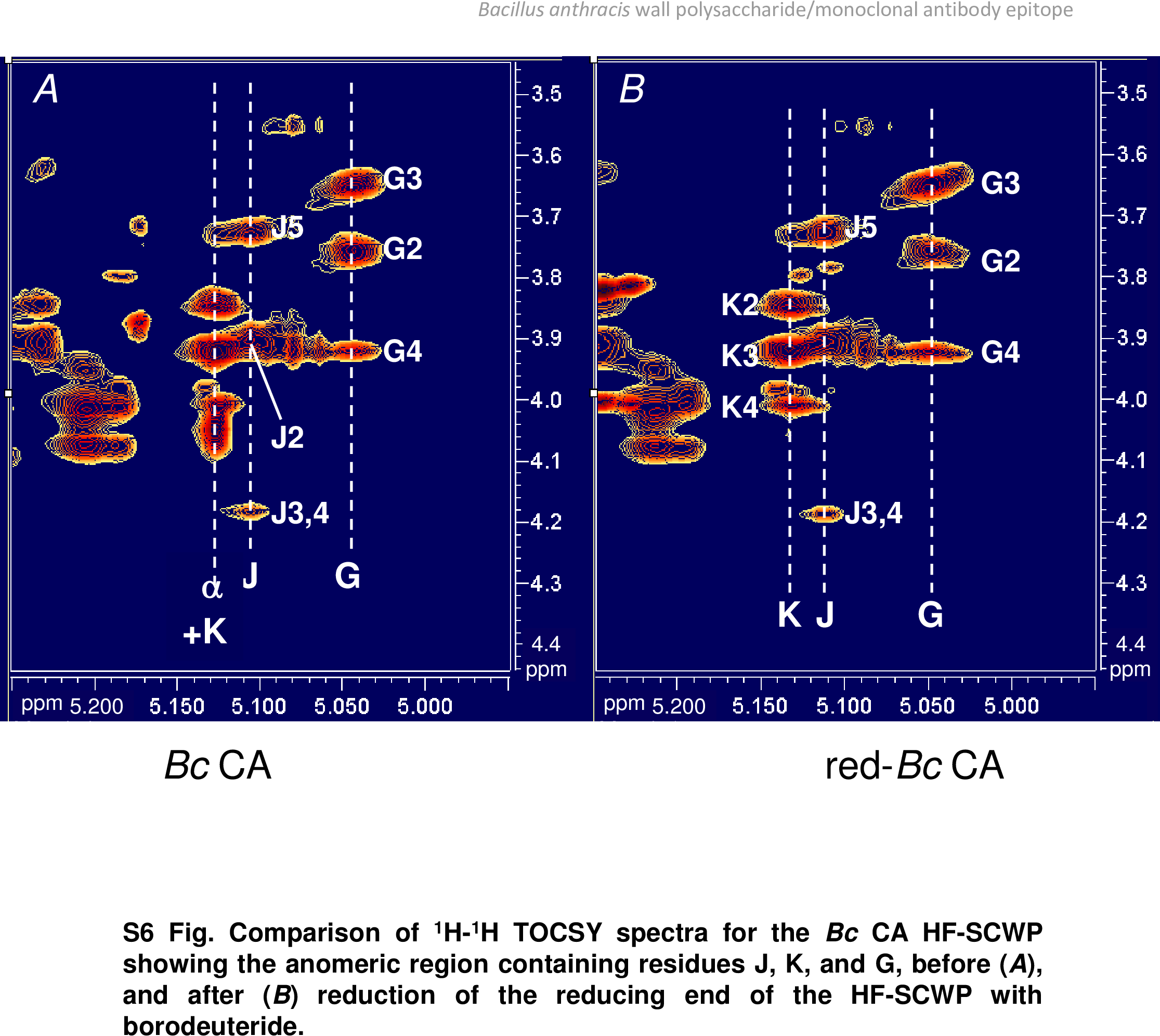

Supplement: S6 Fig — Comparison of 1H-1H TOCSY spectra for the Bc CA HF-SCWP showing the region containing residues J, K, and G, before (A), and after (B) reduction of the reducing end of the SCWP with borodeuteride. In panel A, α = α-reducing end GlcNAc anomeric proton. Certain scalar correlations arising from this α-reducing end anomeric proton overlapped with some of the K residue protons; removal of these α-reducing end signals by borodeuteride reduction allowed definitive assignment of the K ring system (panel B). The β-GlcNAc reducing end anomeric is found at δH 4.74 (refer to S3, S5A, and S8 Figs and the Table 2footnote: “Additional Signals”). (TIF) [file pone.0183115.s006.tif]

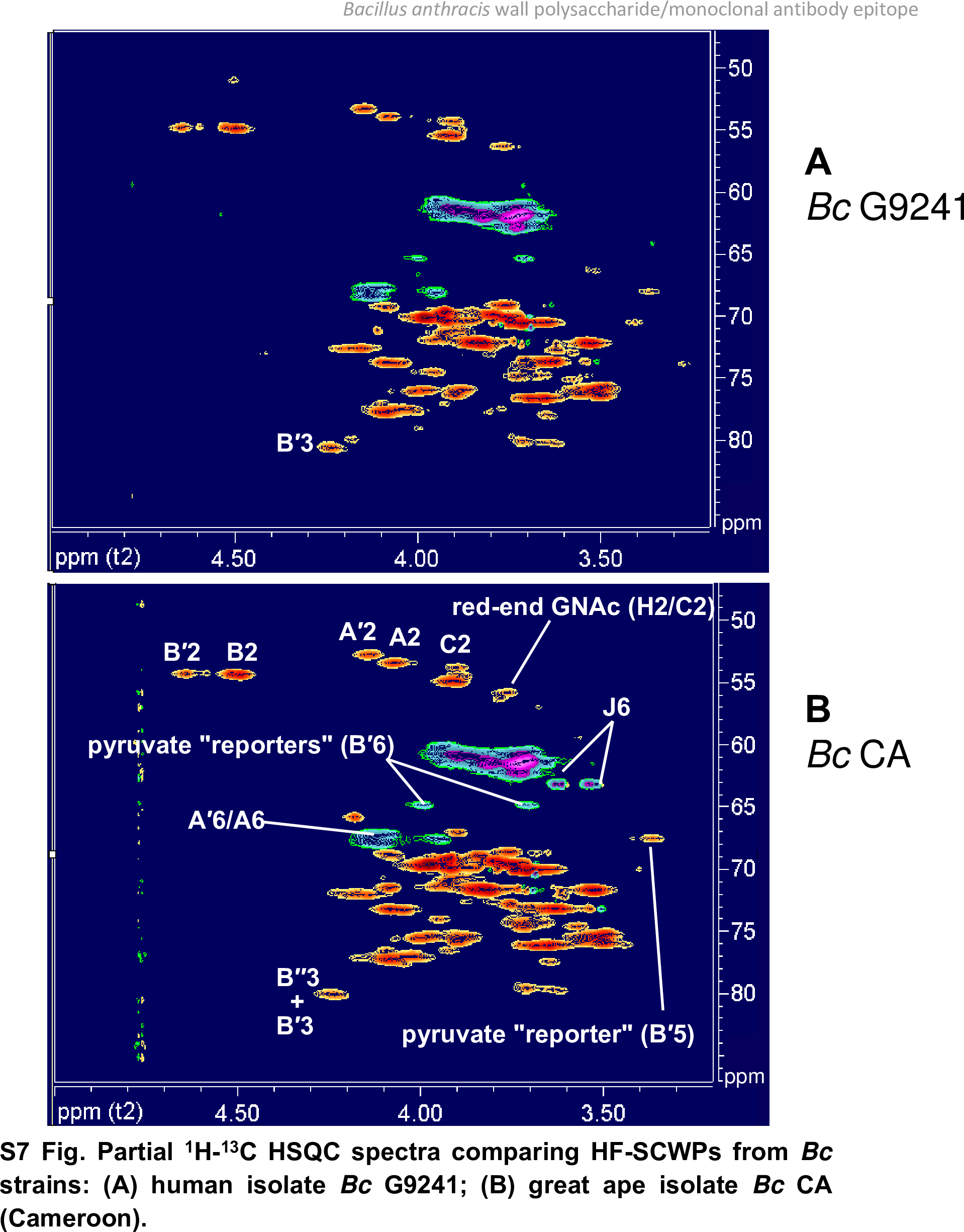

Supplement: S7 Fig — Partial 1H-13C HSQC spectra comparing HF-SCWPs from Bc strains: (A) human isolate Bc G9241; (B) great ape isolate Bc CA (Cameroon). Proton multiplicity is indicated by peak polarity, i.e., orange/red gradient signals (positive polarity) arise from methyl or methine ring protons, and blue/pink gradient signals (negative polarity) arise from methylene (-CH2-) protons. Distinct signals arising from position H6/C6 of residue J (“J6”) were observed in Bc CA (also Bc CI, not shown), which were absent in the human Infection-associated Bc G9241 and Ba strains (previously published [26,27]). In addition, a 1H-13C H2BC analysis provided 2-bond couplings which assisted in residue J assignments (S10 Fig). Pyruvate "reporter signals" and the location of pyruvate in HF-SCWPs from representative Ba and Bc strains is discussed in detail in Forsberg et al., 2012 [26]. Briefly, pyruvate ketal is linked to positions 4 and 6 of the terminal, non-reducing end ManNAc residue, designated B′. This substitution precludes further chain polymerization. At B′ positions 4, 5, and 6, unique δH/δC result from this pyruvate substitution (yielding unique “reporter signals”). A 3-bond 1H-13C HMBC coupling was also observed between the pyruvate C2 carbon, and a favorably oriented H6 proton of B′ for all examined HF-SCWP [26]. These and other reporter signals, arising from substoichiometric modifications (e.g., O-acetylation), occur at virtually identical resonance in HF-SCWPs from all examined strains of Ba and Bc. (TIF) [file pone.0183115.s007.tif]

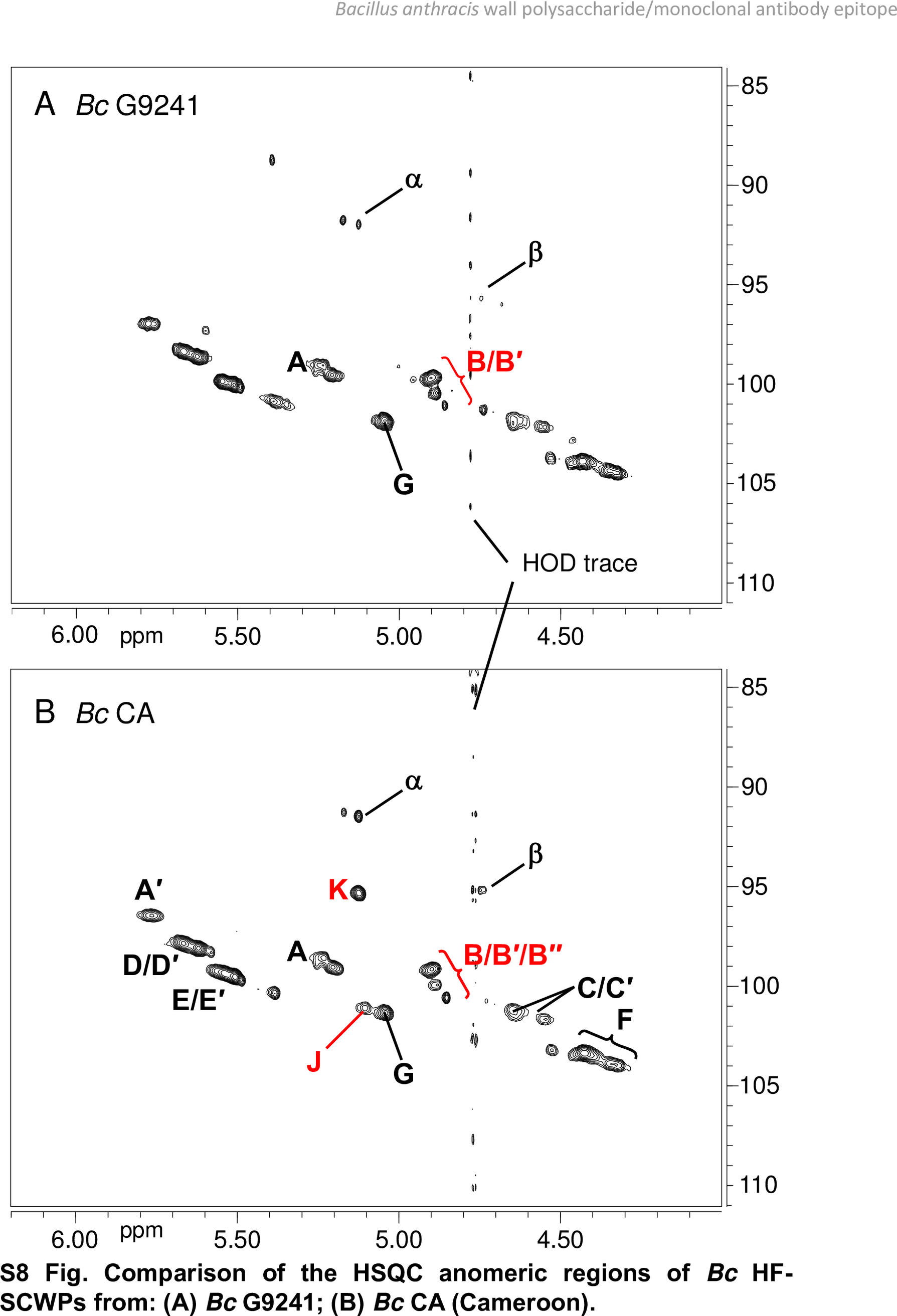

Supplement: S8 Fig — Comparison of the 1H-13C HSQC anomeric regions of Bc HF-SCWPs from: (A) Bc G9241; (B) Bc CA (Cameroon). The spectra are virtually superimposable, with the exception of "new" anomeric signals from residues J and K in the Bc CA sample. Additional heterogeneity of residue B (residue B′′) is also observed in Bc CA (and Bc CI) SCWPs. The α- and β- labels designate the anomeric signals arising from the reducing-end GlcNAc residue of the respective HF-SCWPs. (TIF) [file pone.0183115.s008.tif]

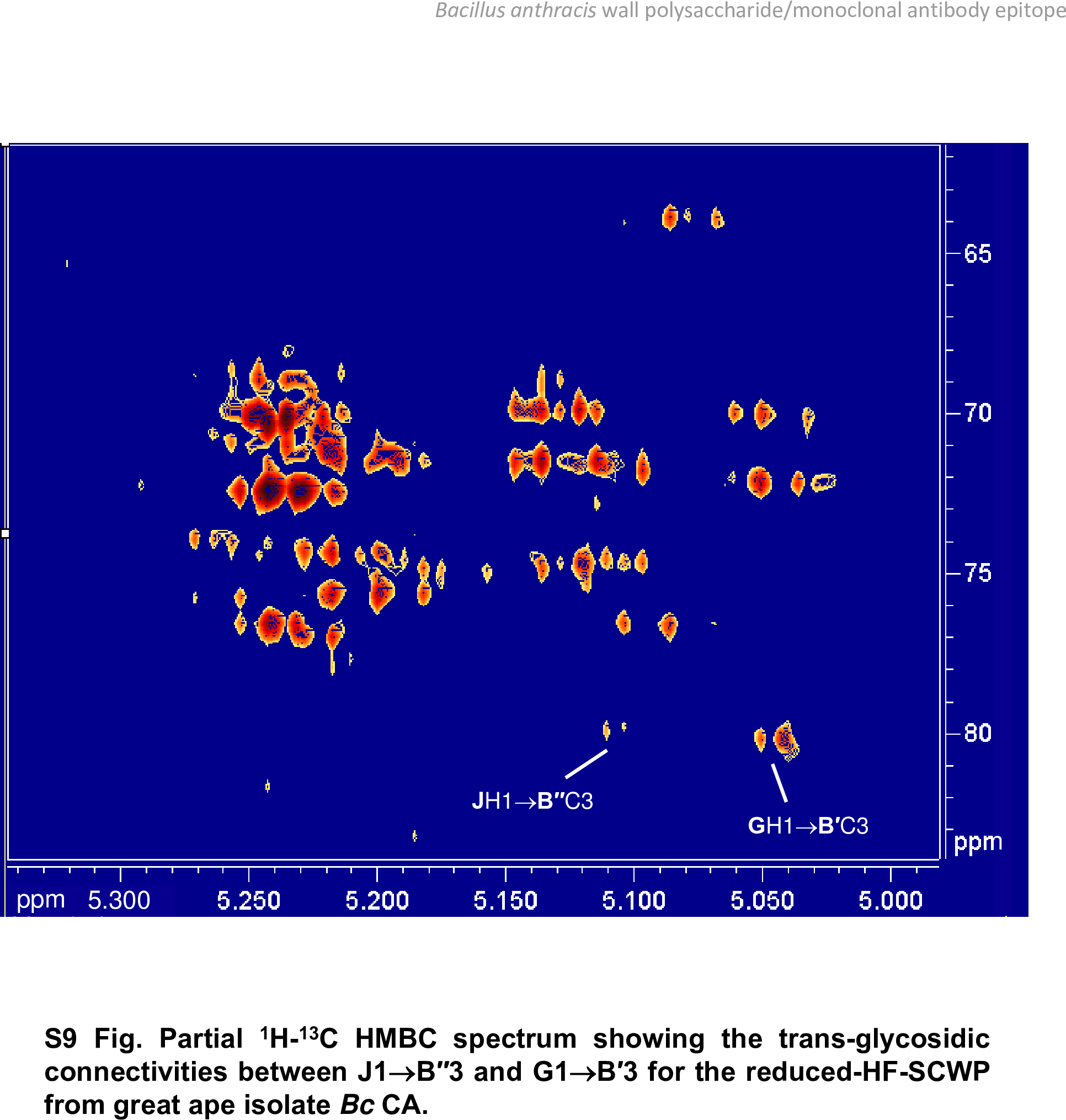

Supplement: S9 Fig — These 3-bond connectivities prove the linkage of residue J to the 3-position of residue B′′, and of residue G to 3-position of B′. (TIF) [file pone.0183115.s009.tif]

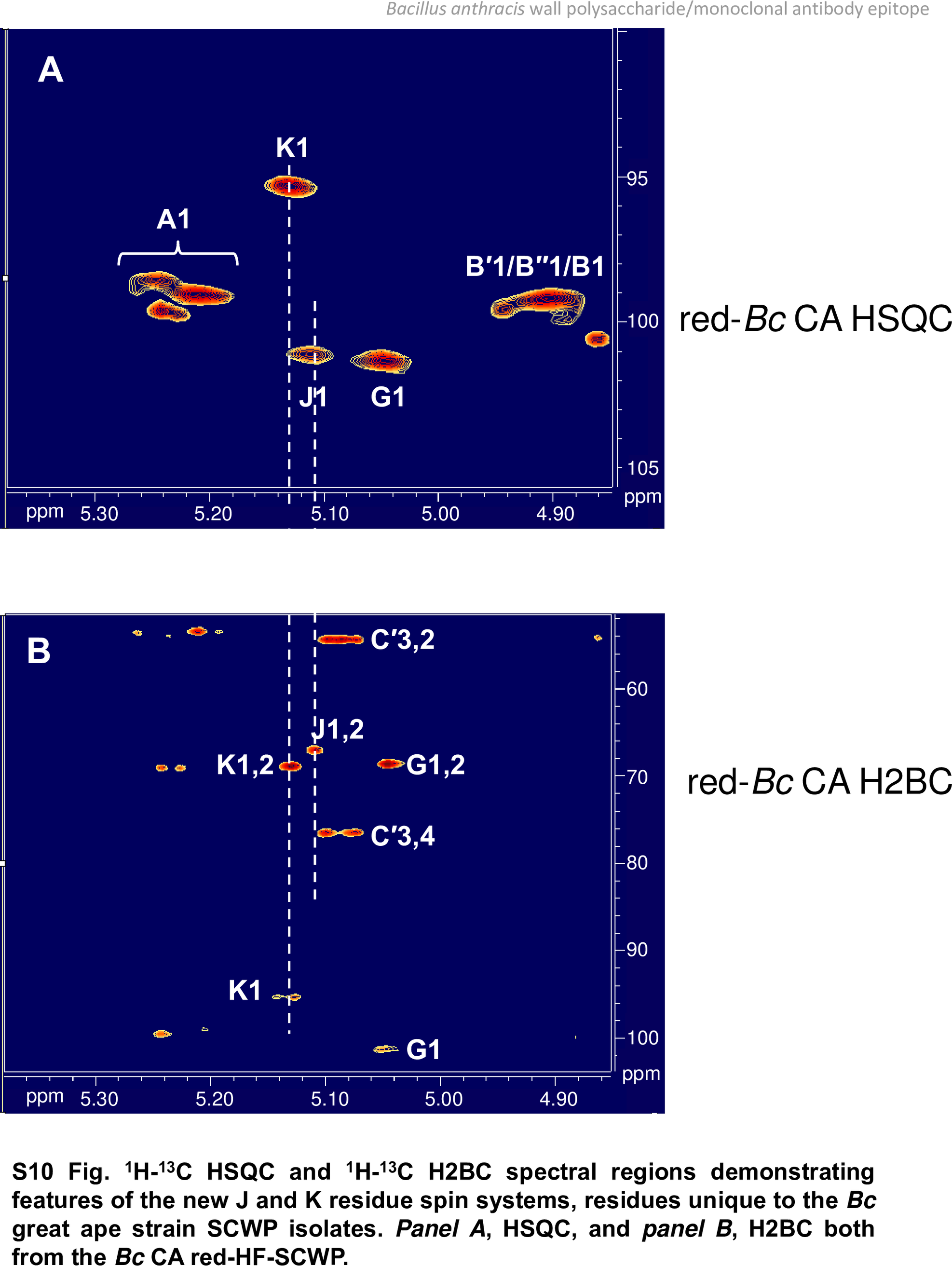

Supplement: S10 Fig — Panel A, HSQC, and panel B, H2BC of the Bc CA red-HF-SCWPs. (TIF) [file pone.0183115.s010.tif]

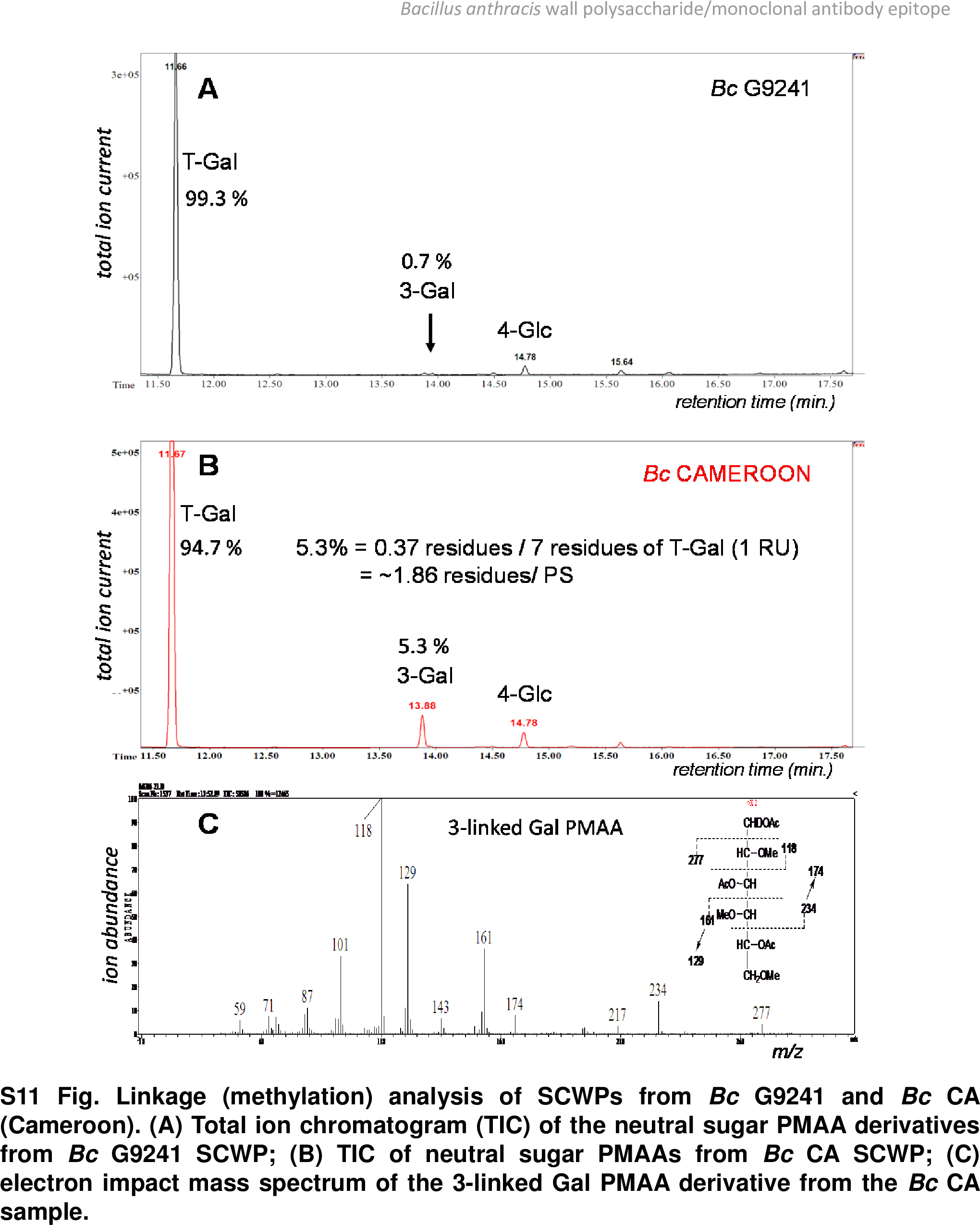

Supplement: S11 Fig — (A) Total ion chromatogram (TIC) of the neutral sugar PMAA derivatives from Bc G9241 SCWP; (B) TIC of neutral sugar PMAAs from Bc CA HF-SCWP; (C) electron impact mass spectrum of the 3-linked Gal PMAA derivative from the Bc CA sample. The GC-MS analysis of the neutral sugar PMAA derivatives demonstrates the presence of 3-linked Gal in the "great ape" strain HF-SCWP, but not in Bc G9241. Only the great ape derived SCWPs (from Bc CA and Bc CI) yield this derivative, which arises from the K1→J3 disaccharide substituent unique to these strains. The results of this chemical analysis (1.86 residues/PS for Bc CA) are in close agreement with NMR signal integration (refer to Table 3). The 4-linked-Glc derivative arises from traces of a contaminating α-glucan, most of which is removed by SEC (see S12 Fig). (TIF) [file pone.0183115.s011.tif]

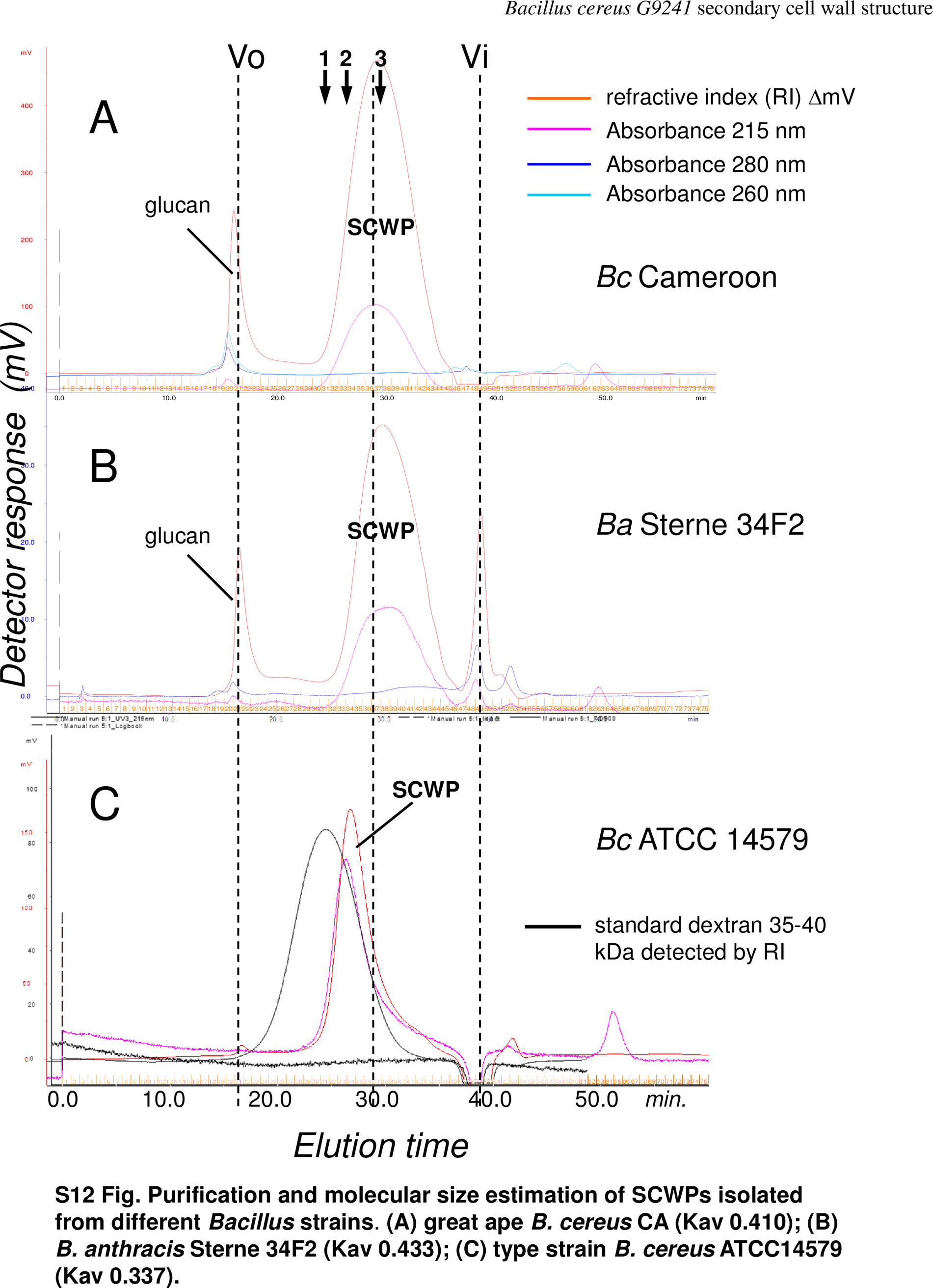

Supplement: S12 Fig — SCWP were released from cell walls by treatment with hydrofluoric acid, dialyzed, then chromatographed on a Superose-12 FPLC size exclusion column with comparison to commercially available α1→6 linked dextrans. A, great ape isolate B. cereus CA (Cameroon) (Kav 0.410). B, B. anthracis Sterne 34F2 (Kav 0.433). C, type strain B. cereus ATCC14579 (Kav 0.337; calculated mass 20,020 Da). The HF-SCWP from the great ape isolates Bc CA/CI and Bc strains all elute at approximately the same location, between the 25 kDa and 10.5 kDa dextran standards, having calculated mass of approx. 12,000 Da for Bc CA (Kav 0.410) and for Bc G9241 (Kav 0.411, not shown). The elution profiles of human isolates Bc G9241 and Bc 03BB87 HF-SCWPs are all essentially identical to that of Bc CA ([27], see Forsberg et al., 2011, Supplement Data for human Bc isolate analysis). Interestingly, the structurally unrelated B. cereus type strain ATCC14579 HF-SCWP elutes slightly after the 25 kDa dextran standard suggesting a mass around 20 kDa. The Bacillus SCWP isolates, in particular those from Ba and pathogenic Bc, contain variable amounts of a high molecular weight glucan which elutes at the void volume (Vo) (panels A and B). Glycosyl composition (GC-MS) and NMR analysis (COSY, TOCSY, HSQC) indicated that this void volume polysaccharide is composed exclusively of glucose in predominately α1→4 linkage. This glucan may correspond to a glycogen polysaccharide previously reported in certain B. anthacis and related pathogenic Bacillus strains. Proteins (i.e., aromatic compounds, Abs 280 nm) also elute at this void region. Column total inclusion volume = (Vi). In addition to the 35–40 kDa dextran profile, the elution positions of the 25 kDa and 10.5 kDa dextrans are indicated by arrows (1 = 35–40 kDa, Kav 0.239; 2 = 25 kDa, Kav 0.299; 3 = 10.5 kDa, Kav 0.433). (TIF) [file pone.0183115.s012.tif]
